# Morphological and physiological adaptations of psychrophilic *Pseudarthrobacter psychrotolerans* YJ56 under temperature stress

**DOI:** 10.1038/s41598-023-42179-x

**Published:** 2023-09-11

**Authors:** Yongjun Son, Jihyeon Min, Yoonjae Shin, Woojun Park

**Affiliations:** Laboratory of Molecular Environmental Microbiology, Department of Environmental Science and Ecological Engineering, Korea University, Seoul, 02841 Republic of Korea

**Keywords:** Bacteria, Microbial ecology, Soil microbiology

## Abstract

Both culture-independent and culture-dependent analyses using Nanopore-based 16S rRNA sequencing showed that short-term exposure of Antarctic soils to low temperature increased biomass with lower bacterial diversity and maintained high numbers of the phylum *Proteobacteria, Firmicute,* and *Actinobacteria* including *Pseudarthrobacter* species. The psychrophilic *Pseudarthrobacter psychrotolerans* YJ56 had superior growth at 13 °C, but could not grow at 30 °C, compared to other bacteria isolated from the same Antarctic soil. Unlike a single rod-shaped cell at 13 °C, strain YJ56 at 25 °C was morphologically shifted into a filamentous bacterium with several branches. Comparative genomics of strain YJ56 with other genera in the phylum *Actinobacteria* indicate remarkable copy numbers of *rimJ* genes that are possibly involved in dual functions, acetylation of ribosomal proteins, and stabilization of ribosomes by direct binding. Our proteomic data suggested that *Actinobacteria* cells experienced physiological stresses at 25 °C, showing the upregulation of chaperone proteins, GroEL and catalase, KatE. Level of proteins involved in the assembly of 50S ribosomal proteins and L29 in 50S ribosomal proteins increased at 13 °C, which suggested distinct roles of many ribosomal proteins under different conditions. Taken together, our data highlights the cellular filamentation and protein homeostasis of a psychrophilic YJ56 strain in coping with high-temperature stress.

## Introduction

Cold environments such as the Arctic Ocean, glaciers, ice caps, permafrost, and sea ice having low temperatures below 5 °C throughout the years account for approximately 80% of the Earth’s environments, which implies their important contribution to the biogeochemical cycle on the Earth^1^. However, relatively low microbial diversity and reduced microbe-mediated ecosystem functions, such as carbon and nitrogen cycles, are prevalent in Antarctic soil due to unfavorable conditions for microbial activity^2^. A substantial variety of bacterial groups (*Actinobacteria, Proteobacteria, Firmicutes,* and *Chloroflexi)* has been discovered in Antarctic regions^3–5^. Notably, distinct clusters belonging to *Actinobacteria* play key roles in ecosystem function, such as global carbon cycling, plant productivity, and bioactive compound production^6^. Interestingly, many *Actinobacteria* genomes also possess genes involved in carbon dioxide fixation through the Clavin-Benson-Bassham (CBB) cycle to gain metabolic energy under limited carbon and nitrogen circumstances^5,7^. All psychrotolerant and psychrophilic *Actinobacteria* including *Arthrobacter* and *Pseudarthrobacter* species must have specific survival strategies to cope with the extreme environmental conditions in Antarctica^3,8^.

Microbes in cold habitats must prevent temperature stress from disrupting enzyme activity and protein stability^9^. One strategy psychrophilic bacteria use to counter this is to upregulate cold-activity enzymes. This increases the composition of unsaturated lipids to improve membrane fluidity, express helicases, cold shock proteins to stabilize DNA/RNA, and utilize carotenoid pigments for protection from solar radiation^10^. *Psychrobacter* sp. PAMC21119 isolated from Antarctic soil upregulated proteins involved in protein folding, metabolite transport, acetyl-CoA metabolism, and membrane fluidity, and downregulated proteins related to heme synthesis, energy conversion, and production in cold environments^11^. *Oleispira antarctica* RB-8 at 4 °C employed the primary protein-folding system by using chaperone Cpn60 as a single heptameric barrel unlike the typical double-barrel structure formed at 16 °C^12^. AtcJ protein of *Shewanella oneidensis*, a functional J-domain protein (JDP) in co-chaperone protein networks, was essential in maintaining proteostasis and bacterial growth at low temperatures^13^. *bgaS* gene encoding *Β*-galactosidase in *Arthrobacter* species isolated from Antarctica was identified as cold-active and increased 5.0-fold at 0–18 °C, in comparison to mesophilic bacteria (such as *Escherichia coli*) at 10–20 °C^14^. Glycine betaine accumulated in cytosol contributed to an improvement in cold tolerance and osmotolerance in psychrophilic bacteria^15^. To increase protein flexibility at low temperatures, proteins of subzero-growing bacteria are composed of more serine and less proline and acidic residues than mesophilic bacteria^16^. *Planococcus halocryophilus* Or1 discovered in Arctic permafrost maintained its cellular energy metabolism and ATP levels through increasing cytochrome C oxidase and ATP synthase at subzero temperature (− 15 °C), the cause of interrupted cellular growth in most bacteria^17^.

The psychrophilic bacteria’s biological activities have been explored through genomic, proteomic, and transcriptomic analyses. These findings indicate that the geochemical carbon cycle can be controlled by bacteria living in cold environments^18^. In Antarctic ecosystems, bacteria play a crucial role in the circulation of carbon, nitrogen, and sulfur by adapting to and occupying extreme environments^19^. Climate change has accelerated the rise in air temperature and sea level and decreased the extent and volume of ice in Antarctica. The global warming emergency causes considerable variation and disruption of microbial communities and activities^20^. To date, scientific data on psychrophiles are unable to sufficiently explain how microbial metabolisms allow for continuous survival and growth in cold environments such as polar regions. Understanding the survival strategies of psychrophilic bacteria can broaden evolutionary and ecological perspectives on microorganisms in Antarctic environments because extremophile bacteria have specific functions allowing them to withstand and grow under harsh stress conditions^5,21^. In this study, bacterial communities in Antarctica soil were acquired to conduct diversity analyses using Nanopore sequencing and terminal restriction fragment length polymorphism (T-RFLP). The complete genome of a dominant psychrophilic bacterium *Pseudarthrobacter psychrotolerans* YJ56 isolated from Antarctica soils was achieved and comparative genomics along with its phenotypic and physiological traits were tested using proteomics under different temperature conditions.

## Results

### The dominance of cold-adapted *Pseudarthrobacter*-like species in Antarctic soil

**Figure 1:**
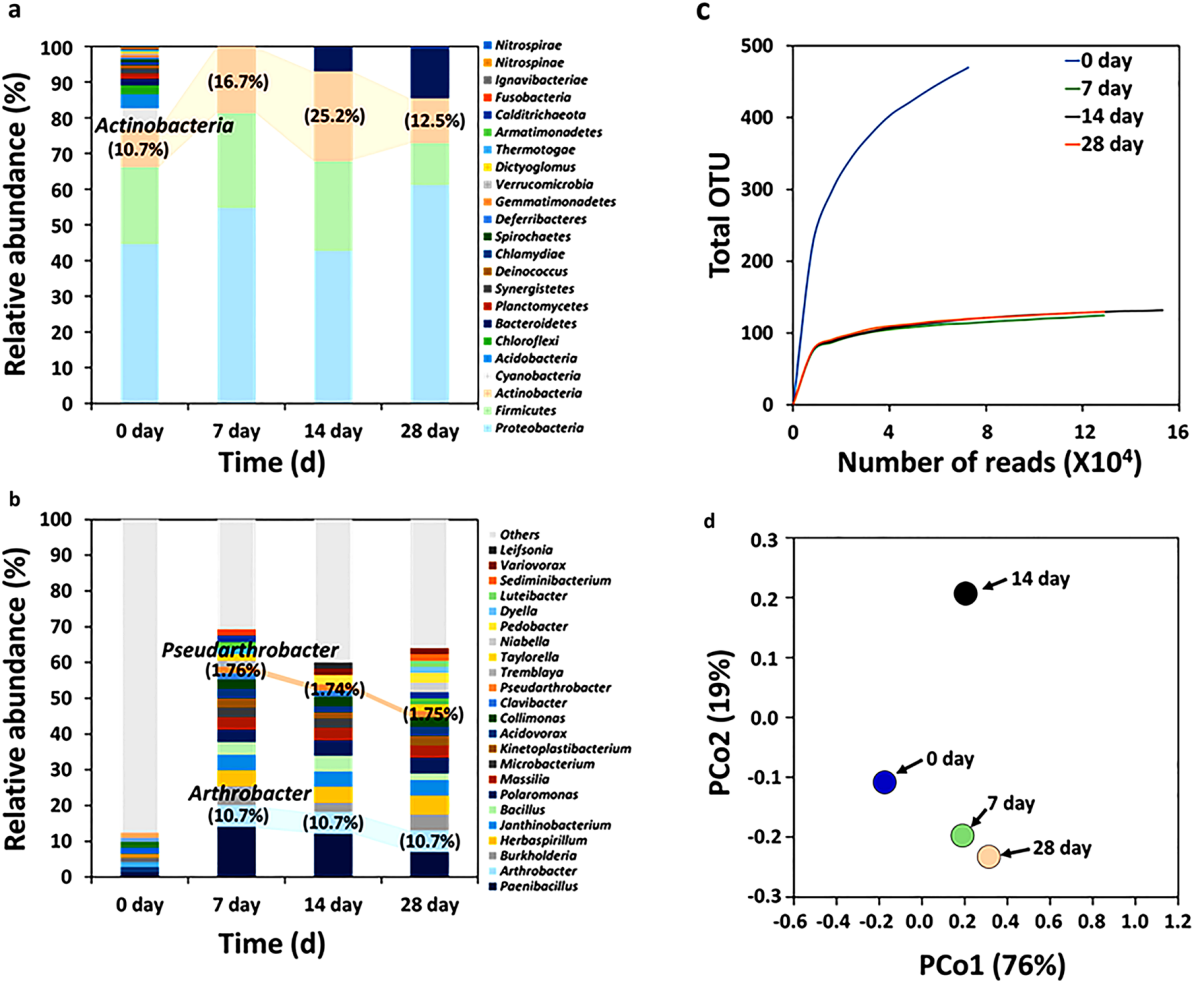
Culture-independent bacterial community in Antarctic soil. (**a**,**b**) The stacked bar plot shows the relative abundance of the bacteria community at the phylum and genus level using Oxford Nanopore sequencing tools. All genomic data were processed using the CLC Genomics Workbench v.21 (Qiagen, Germany) software, based on the NCBI microbial genome database. (**c**) Alpha diversity and (**d**) PCA analyses of bacterial communities were conducted using CLC genomics Workbench (blue color, 0 day incubation of Antarctic soil; green color, 7 days incubation of Antarctic soil; black color, 14 days incubation of Antarctic soil; light yellow color, 28 days incubation of Antarctic soil).

Bacterial community analyses were performed to determine dominant species in Antarctic soil collected from the Cape Burk area. The obtained soil DNA sample was analyzed using the Oxford Nanopore platform based on the full length of the 16S rDNA sequences retrieved from the National Center for Biotechnology Information (NCBI) database. The DNA samples were harvested after specific incubation periods (0, 7, 14, and 28 days) in rich nutrient conditions (R2A) at different temperatures (13 °C vs. 30 °C) with 220 rpm. Psychrophilic bacteria exhibit growth within temperature ranges of 0 °C to 25 °C. Conversely, their growth is impeded at the optimal temperature of 30 °C, which is preferred by mesophilic bacteria^3,16^. In total, 23 phyla (specifically *Proteobacteria, Firmicutes,* and *Actinobacteria*) and 299 genera were identified before incubation (day 0; Fig. 1a,b). Similar bacteria community distributions at the phylum and genus levels were also observed in other Antarctic regions, with *Proteobacteria, Firmicutes,* and *Actinobacteria* being predominant^22^. The diversity of phyla and genera decreased rapidly due to the loss of non-competitive bacteria or obligate anaerobes (e.g., genera *Clostridium, Calditerrivibrio, Desulfotomaculum*, and *Eggerthella*). Interestingly, both genus *Arthrobacter* and *Pseudarthrobacter* belonging to the phylum *Actinobacteria* notably increased after cultivation in R2A at 13 °C, accounting for more than 10.7% and 1.7%, respectively (Fig. 1b). Taxonomic profiles were constructed using the full-length 16S rRNA gene sequences retrieved from the metagenomic dataset, and operational taxonomic units (OTUs) were calculated based on a 90% similarity threshold (Fig. 1c). Our rarefaction analysis suggested that the diversity of our Day 0 sample was much higher than samples from other days (7, 14, and 28), indicating a decrease in bacteria diversity after incubation. A principal coordinate analysis (PCoA), using Bray–Curtis Dissimilarity distances, indicated that all samples (0, 7, 14, and 28 days) clustered separately from our Day 0 (PCoA1:76%) (Fig. 1d), indicating that a taxonomic shift was apparent following incubation in R2A at 13 °C. The bacterial biomass in our sampled Antarctic soil was estimated using DNA yields and bacterial colony-forming units (CFU) (Fig. S1). The amount of extracted DNA per 1 g of dried soil was increased from about 27 to 553 ng/µL from Day 7 to Day 14 and then decreased to 179 ng/µL from Day 14 to Day 28 (Fig. S1a). Moreover, the cell density at 13 °C exhibited an increase from approximately 1.4 × 10^5^ to 1.5 × 10^9^ CFU/mL between Day 0 and Day 14. Subsequently, it decreased to around 1.5 × 10^6^ CFU/mL on Day 28. In contrast, when subjected to R2A at 30 °C, there was a notable decrease in biomass from Days 0 to 7, and there was an absence of colony formation right from Day 0, suggesting that bacteria acquired from Antarctic soil could not grow at higher temperatures (Fig. S1c,d). The cellular abundance of *P. psychrotolerans* YJ56 in Antarctic soil was also observed by our T-RFLP analysis utilizing restriction enzymes (*MspI* or *Rsal*). A psychrophilic *P. psychrotolerans* YJ56 strain was one of the initial dominant bacteria with a remarkable growth rate under R2A at 13 °C (Fig. S2a,b). The peak height value of strain YJ56 increased when the Antarctic soil was enriched in an R2A medium at 13 °C for 7 days. However, the population of strain YJ56 was gradually diminished from Days 14 to 28. Our DNA-based analyses demonstrated that both *Arthrobacter* and *Pseudarthrobacter* genus from Antarctic soil were dominant psychrophilic groups in our laboratory cultivation conditions.

### Isolation of psychrophilic *Pseudarthrobacter psychrotolerans* in Antarctic soil

**Figure 2:**
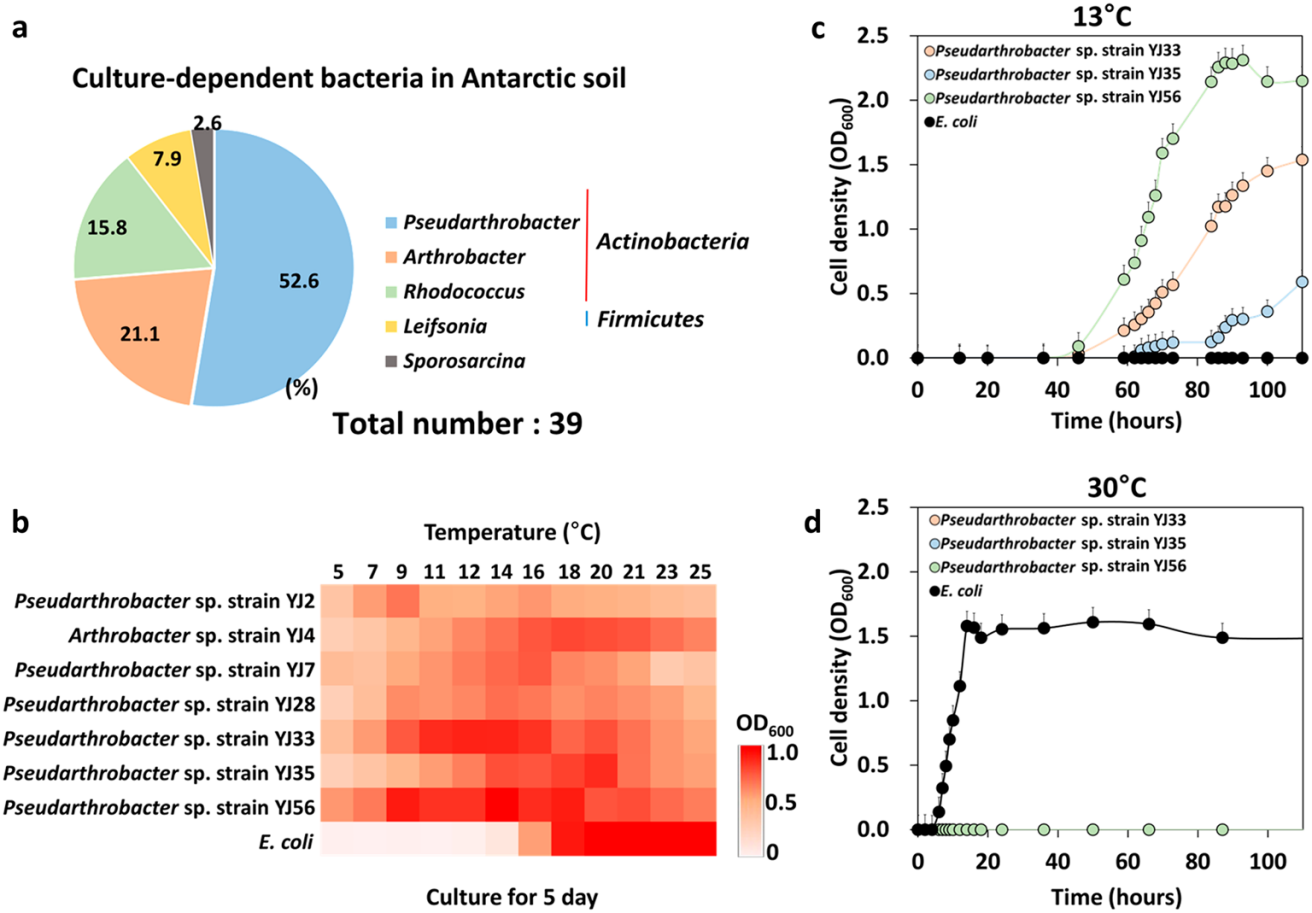
Culture-dependent bacterial communities in Antarctica soils. (**a**) The bacterial community analysis was performed using 16S rRNA sequence genes from 39 strains isolated from Antarctic soil enriched in an R2A liquid medium at 13 °C for 5 days. All values were described as a percentage (%). (**b**) Psychrophilic strains isolated from Antarctic soil were cultured for 5 days from 5℃ to 25 °C. The strains were cultured for around 5 days at (**c**) 13 °C or (**d**) 30 °C, respectively. The bacterial growth levels were measured by OD_600_ value. *E. coli* ATCC 25922 as a mesophilic bacterium was used for comparison with other *Pseudarthrobacter* species on bacteria growth depending on temperature. The initial inoculum was 1 × 10^6^ CFU/mL. All experiments were performed in triplicate and standard deviations were indicated by error bars.

**Figure 3:**
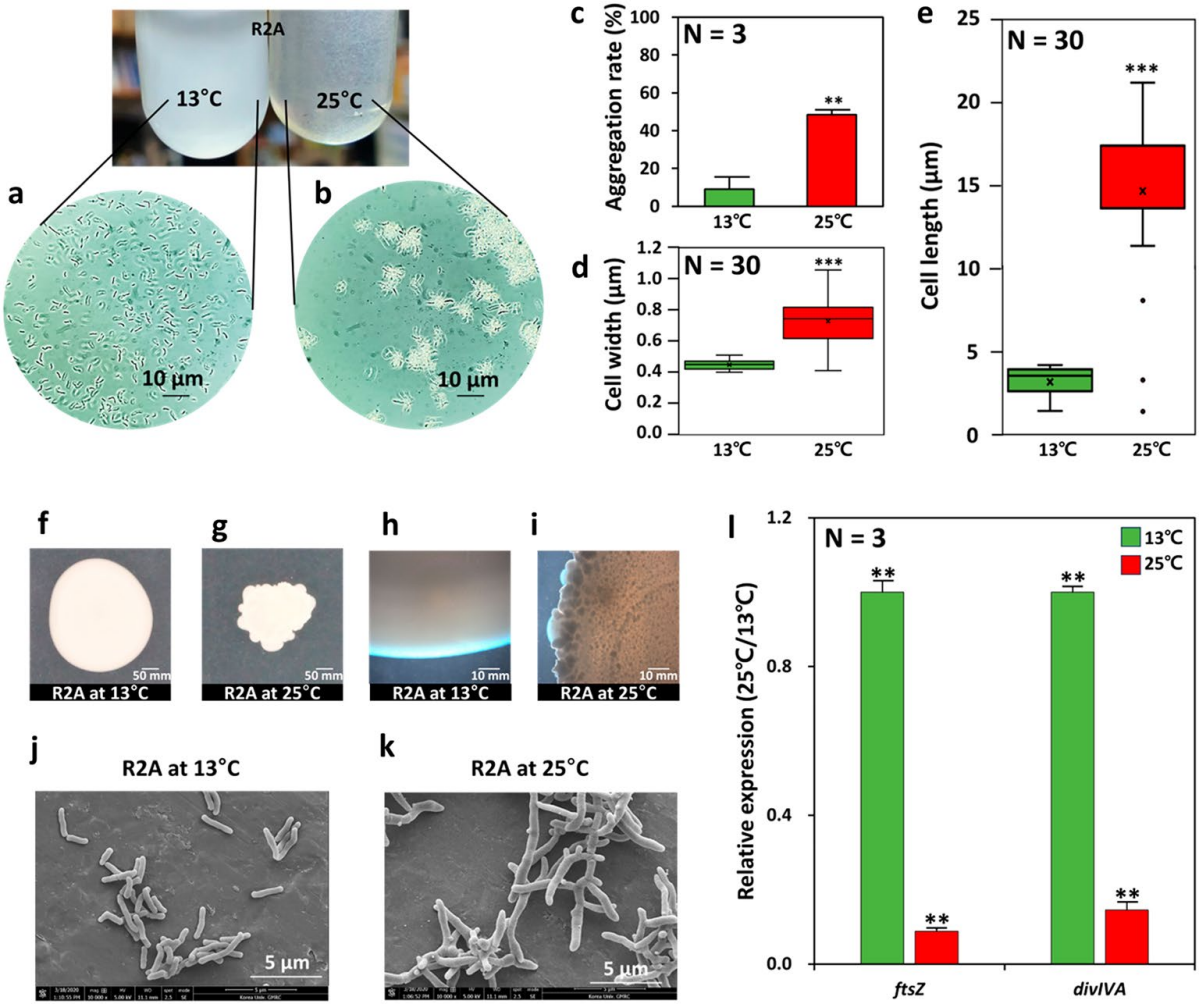
Cellular morphology and relative gene expression of strain YJ56 cultured on an R2A liquid medium at different temperatures (13 °C and 25 °C) until the exponential phase (OD_600_: 1.0). (**a**,**b**) Observation by using phase-contrast microscopy (× 1,000 magnification) with 10 μm of scale bar, and (**c**) measurements of cellular aggregation rate of strain YJ56 at different temperatures (**d**,**e**) Evaluation of different cellular length and width of strain YJ56 at different temperatures. Cellular colonies of strain YJ56 formed on R2A agar plates at (**f**,**h**) 13 °C, or (**g**,**i**) 25 °C for 14 days. (**f**,**h**) observation by using a stereomicroscope (× 4 magnification) with 50 mm of scale bar, and (**g**,**i)** inverted microscopy (× 1000 magnification) with 10 mm of scale bar. (**j**,**k**) observation by SEM (× 10,000 magnification) with 5 μm of scale bar. (**l**) Expression of the *ftsZ* and *divIVA* genes in strain YJ56 strain under 13 °C and 25 °C. Error bars indicate standard deviations and asterisks (*) indicate significant differences between data of 13℃ and 25 °C conditions, according to the *t*-test (*p < 0.1, **p < 0.05, and ***p < 0.001).

Among the 60 strains isolated from Antarctic soil, a total of 39 strains were chosen as the bacterial groups with superior growth (OD_600_ > 1.0) under low temperatures (13 °C) within 5 days of inoculation (Fig. 2a, Fig. S3). Culture-dependent bacterial identification using the 16S rRNA sequence was performed on these 39 strains (Table S1). The phylum *Actinobacteria* including *Pseudarthrobacter*, *Arthrobacter*, *Rhodococcus*, and *Leifsonia* species was dominantly present as it accounted for 97.4% of the total community population. The genus *Pseudarthrobacter* (21 strains) was dominant, accounting for 53.8% of cultured bacteria isolated from the Antarctic soil. (Fig. 2a, Table S1). The genus *Arthrobacter* was the second major psychrophilic bacteria (20.5%), followed by the genus *Rhodococcus* (15.3%)*, Leifsonia* (7.6%), *and Sporosarcina* (2.8%). Seven psychrophilic bacteria including *Pseudarthrobacter* and *Arthrobacter* species were selected because they achieved a high cell density (OD_600_ ~ 3.0) under our tested conditions (Fig. S3). The optimal growth temperatures for these seven strains were further verified through incubation at 5 °C to 25 °C with PhotoBiobox for five days without shaking (Fig. 2b). Three *Pseudarthrobacter* strains (YJ33, YJ35, and YJ56) reached a higher cell density (OD_600_ > 1.0) within these five days than other strains at low temperatures (< 16 °C). *Pseudarthrobacter* sp. strain YJ56 was chosen for additional study because of its superior growth capability at 13 °C compared to all other tested strains (Fig. 2c). Taxonomic characterization using the 16S rDNA information and other necessary assays named the strain YJ56 as *P. psychrotolerans* YJ56, a novel cold-adapted bacterium^23^. Three psychrophilic bacteria were able to grow at 13 °C, but not at 30 °C (Fig. 2d)*.* Interestingly, lowering growth temperature allowed YJ56 cells to grow at 25 °C, but a dramatic morphological change (from rod shape to branched filaments) was noticeable by their buoyancy as cell aggregates in the liquid culture (Fig. 3a,b). Aggregation rates were quantified using a mild centrifugation step (2,000 rpm), specifying that much a higher aggregation percentage (54.5 ± 2.4%) was monitored at 25 °C compared to cells grown at cold temperatures (14.5 ± 3.2%) (Fig. 3c). Consistent with these observations, cellular sizes of the YJ56 strain cultured at 25 °C were wider and longer (widths: 0.6–0.9 μm, lengths: 11.1–18.2 μm) than cells at 13 °C (widths: 0.4 μm, lengths: 2.7–3.0 μm,) (Fig. 3d,e, Fig. S4). The edges of a colony grown on an agar surface at 13 °C exhibited a circular and smooth appearance (Fig. 3f). However, the colony morphology of cells at 25 °C had irregular shapes with a rough surface, probably due to aggregated branching cells on the agar plates (Fig. 3g). Both colonies were magnified by stereomicroscope (× 1000 magnification) (Fig. 3h,i), providing the observation that the colony of strain YJ56 had rough margins with a multitude of dark dots under heat stress conditions. The SEM image analysis clearly depicted the branching formation of heat-stressed YJ56 cells (Fig. 3j,k). Analysis of our data unveiled that the decrease in expression of the *ftsZ* and *divIVA* genes (0.08 and 0.01-fold, respectively) at 25 °C might lead to a filamentous phenotype marked by insufficient cellular separation (Fig. 3l)^24^. Our observational and qRT-PCR data proved that continuous heat stress led to abnormal cellular morphology and a rough colony phenotype for psychrophilic strain YJ56.

### Comparative genomics of *P. psychrotolerans* and other *Pseudarthrobacter* species

**Figure 4:**
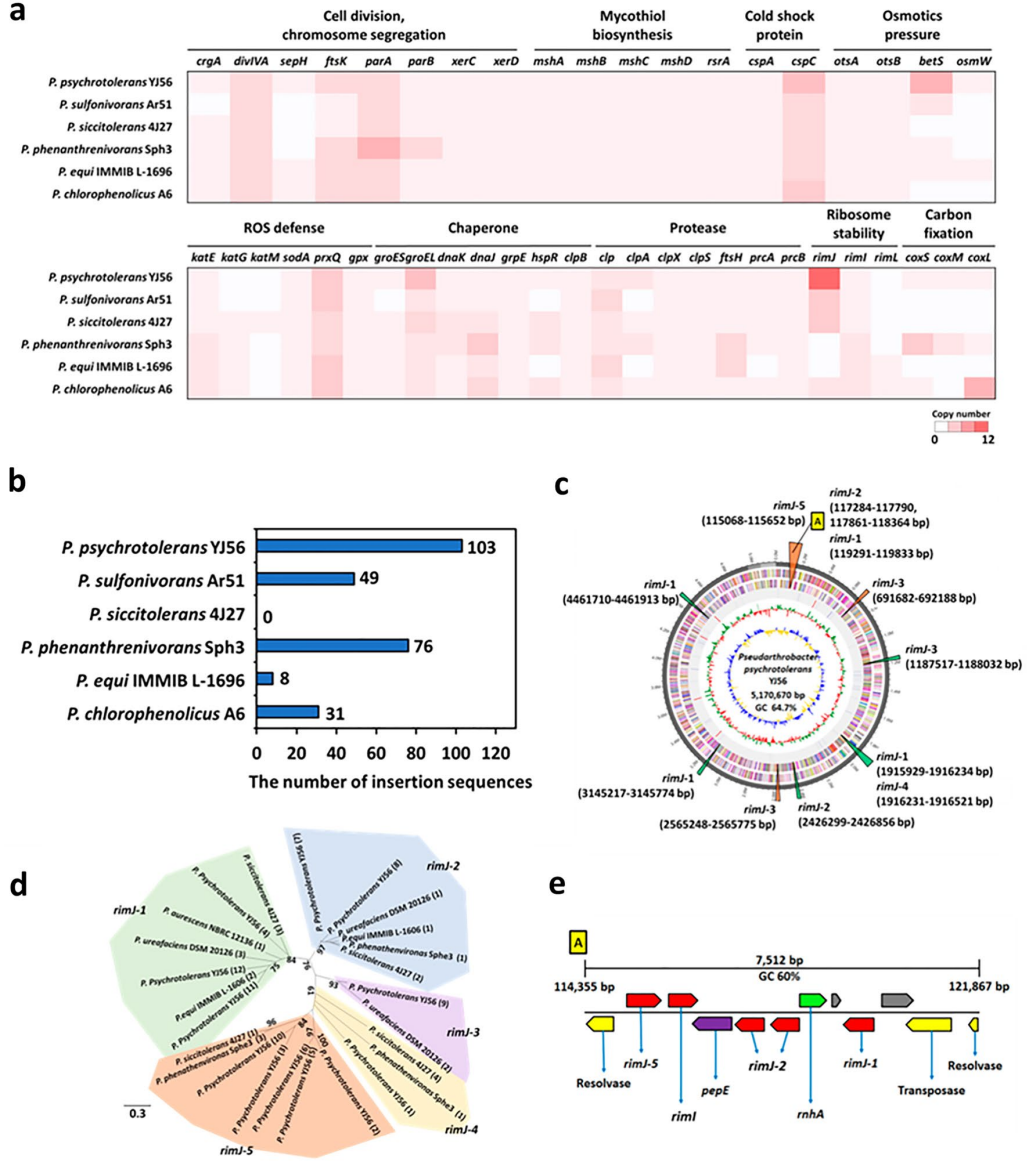
Genomic analysis of strain YJ56. (**a**) A heap map showing the gene copy number of *P. psychrotolerans* and its closest strain. (**b**) The number of insertion sequences in *Pseudarthrobacter* species. The complete genomic sequences of each strain were analyzed to evaluate the number of insertion sequences. Insertion sequences with *E*-value ≥ 0.0001 were counted. (**c**) Distribution of *rimJ* gene in the entire genomic sequence of strain YJ56. The genomic sequence comprises 5,170,670 bp with 12 copies of *rimJ* gene and 64.7% of GC ratio. Green triangles indicate six copies of *rimJ* gene sequences identified in both the strain YJ56 and other *Pseudarthrobacter* species. Orange triangles indicate six copies of *rimJ* gene sequences identified in only strain YJ56 rather than other *Pseudarthrobacter* species. The “A” in the yellow box represents the site with relatively abundant *rimJ* gene copies among specific contained genomic sequences in strain YJ56. (**d**) Sequence similarity between *rimJ* genes in the entire genomic sequence of strain YJ56 and other *Pseudarthrobacter* species. The *rimJ* genes with more than 30% of sequence similarity were grouped into *rimJ-1–5*. The phylogenetic tree was constructed using maximum-likelihood algorithms. (**e**) The “A” site with abundant *rimJ* copies in the entire genome sequence of strain YJ56. The scale bar indicates the size of the total base pair in the site with 60% of GC ratio. Each identified gene and protein is depicted using a specific color, and the hypothetical protein was classified with gray.

Gene synteny and comparative genomics analyses were performed between strain YJ56 and the phylogenetically related five *Pseudarthrobacter* species (Fig. S5a,b). Strain YJ56 has the largest genomes (~ 5.17 Mbp) and high GC contents (64.7%) amongst all other *Pseudarthrobacter* species [genome sizes (4.39–4.77 Mbp), GC contents (64.7–66.3%)] (Table S2). Its largest genome is attributed to the presence of many IS elements (n: 103, ~ 2.4% of the genome, e.g., IS1380, IS701, Tn3 family transposons) as well as functional genes such as *cspC*, *groEL, bet*S, *rimJ* genes linked to stress defense and ribosome stability (Fig. 4a,b, Table S2). Syntenic analyses of our tested six *Pseudarthrobacter* genomes revealed that ortholog gene clusters (n: 2107) involved in cell division machinery (*divIVA, ftsK, parAB,* and *xerCD* genes) and the mycothiol biosynthetic pathway (*mshABCD* and *rsrA* genes) were conserved, which are only found in the phylum of *Actinobacteria*^25,26^ (Fig. 4a). Particularly, the psychrophilic YJ56 strain harbors diverse unique gene clusters (n: 68) associated with cellular adaptation to cold shock and low nutritional conditions (Table S3). Some transcriptional regulators including SrmB, DbpA and DeaD belonging to a DEAD/DEAH box helicase family protein have high identities (58.0–80.8%) with those of *Arthrobacte*r sp. 31Y and appeared to function for the assembly of 50S ribosomal proteins and RNA folding at low temperatures^27^. The highly efficient uptake of compatible solutes in strain YJ56 at low temperatures may be expected due to six copies of *betS* along with one copy of *opuD* compared to the low copy number of *betS* (0–2 copies) in other *Pseudarthrobacter* strains, providing bacterial cells with increased uptake capacity for stress-relieving solutes (e.g., glycine, betaine, proline) under both salt and osmotic stress conditions^28,29^.

Comparative genomic analysis showed different distributions of oxidative stress-defense genes (*kat*, catalase; *sod*, superoxide dismutase; *prx*, peroxidase; *gpx*, glutathione peroxidase), major chaperone genes (*groEL* and *dnaK*), and proteases (*clp* genes) (Fig. 4a). Different degrees of cellular adaptation genes under high or low-temperature conditions could be presumed, suggesting that phenotypic alteration of strain YJ56 might contribute to survival strategies in hostile environments. The presence of multiple chaperones (5 copies of the *groEL* gene, nucleotide similarity: ns, > 92.1%), proteases, and peroxiredoxins (4 copies of the *prxQ* genes, ns. > 95.4%) suggested the continuation of endogenous cellular stresses during the growth and metabolisms of psychrophilic cells (Fig. 4a). Interestingly, strain YJ56 cells possess a higher number of *rimJ* genes (12 copies vs. 3–4 copies in other *Pseudarthrobacter* strains), encoding ribosomal-protein-S5-alanine N-acetyltransferase which functions as a ribosome assembly factor (Fig. 4c). This might contribute to translation fidelity and psychrophilic traits during growth at cold temperatures^30^. The non-identical *rimJ* genes with more than 30% of nucleotide similarity can be clustered into five groups (*rimJ-1*–*5*) based on their phylogenetic relationships (Fig. 4d). Given their overall low nucleotide similarity and multiple copies of RimJ in the same clusters, the *rimJ* genes might expect as different expression patterns by various environmental stimuli and distinct roles. In uropathogenic *E. coli*, the RimJ controls the expression of the *papBA* transcription for P pili production although the underlying mechanisms of multiple RimJ remain unclear^31^. Interestingly, the region A (114,355–121,867 bp, marked with “A” in Fig. 4e) has multiple *rimJ* genes and only one *rimI* gene encoding a ribosomal-protein S18 alanine N-acetyltransferase in short distances with a 60% GC ratio. RimI with acetylation activity targeting two distinct objectives [ribosomal protein S18 and elongation factor Tu (EF-Tu)] was also known for high translation efficiency in *E. coli*, which might be also true to our strain^32^. Low GC ratios (60.0% vs. 64.7%, chromosomal backbone) and three genes linked to both transposase and resolvase, suggest a high possibility for horizontally transferred *rimJ* genes in region A (5 copies) (Fig. 4e). These transposase genes are identical (100%) with IS427 found in many other bacterial species (*Mycobacterium tuberculosis*, *Streptomyces coelicolor*, *Azospirillum* sp. B510, and *Rhizobium etli*) (Table S4). Taken together, our data revealed that strain YJ56 has a variety of unique gene clusters and multiple cold-adaptation-related genes (*cspC, betS, prxQ,* and *groEL* genes) to survive under harsh environments.

### Abundance of chaperones at proteomic analysis under high temperatures

Proteomic analyses using bacterial cells grown at 13 °C and 25 °C were conducted to identify important proteins for cold or hot temperature adaptation (Fig. S6). Among all identified spots (n: 503), paired spots (green, n: 238) and non-paired spots (red, n: 265) were observed under both temperature conditions (13 °C or 25 °C) (Fig. S6). The most abundant spots (6, 13 °C; 10, 25 °C) with high fold level (≥ 2.0-fold) and unique spots (4, 13 °C; 9, 25 °C) were also noted (Fig. S7). Different degrees of protein levels involved in ribosome assembly (e.g., S4 and S10, 25 °C; L18 and L29, 13 °C) were noticeable, which might be linked to the stability or translational capacity of ribosomes at different temperatures (Table S5). However, we cannot rule out the possibility that these ribosomal proteins might have unknown moonlighting functions by interacting with other cellular proteins for temperature adaptation^33^. At 13 °C, chromosome partitioning, with ParB functioning as a division regulator, was significantly highly produced (2.7-fold) when compared to 25 °C. This observation indicates that low levels of ParB proteins at 25 °C might lead to bacterial filamentation by inhibiting chromosome segregation and cell division^34^. Consistent with other reports, two chaperone GroEL proteins were extremely overproduced (2.1 to 7.0-fold) at 25 °C, which may aid protein refolding and prevent aggregation of misfolded polypeptides under high-temperature conditions^35,36^. Interestingly, our proteomic data showed that two proteins (heat-stable leucyl aminopeptidase PepA and catalase KatE) were responsible for protein homeostasis and oxidative stress-defense, respectively. As they were only detected at 25 °C, this theorizes that the psychrophilic strain YJ56 experienced heat-induced protein misfolding and cellular oxidative stress under 25 °C^37^. We acknowledge a weak molecular linkage between our proteomic data and cellular filamentation, except in the case of ParB. However, it is worth noting that many stress-related proteins, including the aforementioned proteins (GroEL, KatE) might be indirectly connected to the dysfunction of cellular division and consequent branched filamentation^37–40^.

## Discussion

Many psychrophilic bacteria employ cold-adaptive mechanisms with a variety of unique genes involved in stress defense systems, nutrient transporters, and secondary metabolite biosynthesis that increase their competitive fitness in a bacterial population^25^. Bacterial filamentation including conditional branch formation is often considered as their adaptive trait and occurs by inhibiting cell division through the loss of septum formation under non-optimal conditions (Fig. 3). The psychrophilic strain YJ56 formed irregularly-shaped colonies with granules on the agar plate and underwent cellular branch formation from steady-state heat stress (Fig. 3). Our proteomic data showed an upregulation of chaperones (GroEL) and catalase (KatE) proteins in the strain YJ56 cultured at 25 °C, suggesting considerable heat and ROS stresses (Table S5). When observing *E. coli* under heat stress, a consequent reactive oxygen species (ROS) generation triggered an SOS response and resulted in cellular elongation with lower growth rates with the action of derepressed SulA, an inhibitor of FtsZ, by the RecA-LexA mediated SOS systems^41,42^. However, this canonical SulA is only conserved in γ-proteobacteria, not in the Actinobacterial group including strain YJ56^42^. Unlike the actin-like MreB-dependent cell shape formation of *E. coli,* the unique cell divisome apparatus (e.g., DivIVA, OtsA, ParAB) controlling apical-basal cell division polarity and shape maintenance is also present in the genome of strain YJ56 (Fig. 4a). Interestingly, OtsA, originally recognized as a trehalose-6-phosphate synthase in non-*Arthrobacter* species, could be involved in a divisome complex of *Arthrobacter* sp. A3 and its low expression or depletion led to the induction of multicellular and myceloid-form cells^43^.

It has been widely known that bacteria within the phylum *Actinobacteria* thriving in harsh environments (cold, low nutrients, osmotic pressure, etc.) are dominant and contribute substantially to ecosystem functions in Antarctic soil, thus their genomic information provides us more insights into their persistence and evolution^3,4^. However, genomic data (a total of 13 species) of *Pseudarthrobacter* species are exceedingly rare and our genomic data of the psychrophilic strain YJ56 was, as far as we know, the first psychrophilic genome at the time of writing. Both metagenome and metatranscriptome analyses of Antarctic soils suggest that the robustness of the Actinobacterial group could be possible because they harbor a relatively high abundance of the *coxSML* genes encoding CO dehydrogenase and the *rbcLS* genes encoding a ribulose-1,5-bisphosphate carboxylase/oxygenase (RubisCO). This sustains the energy and carbon source required through scavenging trace gasses including CO_2_, CO ubiquitously distributed in the atmosphere^4^. Our psychrophilic strain YJ56 also possesses these *coxSML* genes, providing a clue to its high sustainability in the poor organic carbon condition of Antarctic soil (Fig. 4a). Prevalence and upregulation of major cold shock proteins (e.g., CspA, CspC, and CspE) and multifunctional RNA binding proteins, could facilitate transcription and translation at low temperatures by functioning as an RNA chaperone and controlling mRNA-mediated protein secretion systems^17,44^. Our genomic data revealed that the psychrophilic strain YJ56 has six copies of the *csp* genes (five *cspC*, one *cspA* genes) in comparison to the low copy number of *csp* genes (one *cspA*, 0–4 *cspC* genes) in five mesophilic *Pseudarthrobacter* species and *E. coli* strains (Fig. 4a).

Another critical machinery for the accumulation of compatible solutes for osmotic homeostasis and freeze-protection in Antarctic soil might be attributed to the six copies of *betS* gene encoding a glycine betaine/proline transporter and one copy of an *osmW* gene encoding an osmoprotectant import permease in the YJ56 strain^45–48^. Other mesophilic *Pseudarthrobacter* species and *E. coli* strains harbor a low copy number of *betS* genes (0–2 *betS*) and lack the *osmW* gene excepting *P. chlorophenolicus* A6 (one copy of the *osmW* gene) (Fig. 4a). In the psychrotrophic bacterium *Nesterenkonia* sp. AN1, both the *opuABCD* and *betAB* genes were commonly upregulated at low temperatures, indicating that they could sustain osmotic homeostasis by using multiple osmolyte uptake systems for compatible solutes (e.g., glycine, betaine, proline)^49,50^. A typical cold-adaptation strategy of psychrophilic having higher proportions of branched and unsaturated fatty acids to improve membrane fluidity was also observed in the strain YJ56 (Fig. S8, Table S6). Taken together, our observation showing high temperature-mediated atypical branch formation of the psychrophilic *P. psychrotolerans* YJ56 along with its genomic data provides the molecular basis for its survival strategies of psychrophilic *Pseudarthrobacter* species in cold environments. Underlying mechanisms for this branch formation remain unclear, particularly how the Actinobacterial group senses heat stress and changes its cell morphology from rod cells to a filamentous shape under non-optimal temperature conditions. Thus, further research on how alterations of membrane and peptidoglycan structures are connected to the repression of cell division and the following branch formation is warranted.

## Methods

### Bacterial isolation from Antarctic soil

Soil samples were collected from the Cape Burk area (1: 74° 45′ 19.6″ S, 136° 48′ 44.6″ W)^51^. The soil (1 g) was inoculated in a Reasoner’s 2A (R2A) liquid (composed of 0.5% proteose peptone, 0.05% casamino acid, 0.05% yeast extract, 0.05% dextrose, 0.05% soluble starch, 0.03% dipotassium phosphate, 0.005% magnesium sulfate, and 0.03% sodium pyruvate, % w/v, pH 7.0) with cycloheximide (100 µg/mL) to suppress the growth of eukaryotic organisms and then cultured at 13 °C with 220 rpm of shaking. The culture was serially diluted to 10^–4^ using phosphate-buffered saline (PBS) after 7 days. Next, 200 μL of each dilution was streaked onto the R2A agar plate diluted to 10^–1^ before culturing the agar plates at 13 °C for 7 days. We randomly selected and isolated 60 colonies from the collective on the R2A agar plates to find psychrophilic bacteria. All the selected strains were cultured for 16 h. Following this, each cell culture [optical density 600 (OD_600_): 0.8] was inoculated into R2A liquid (5 mL) and cultured for 5 days at 13 °C and 30 °C with 220 rpm of shaking, respectively. The OD_600_ value was measured using a spectrophotometer (Eppendorf, Germany) to distinguish bacteria with a high growth rate among bacteria cultured at 13 °C, but not at 30 °C. Then, bacteria with a superior growth rate at 13 °C were cultured for 5 days using the PhotoBiobox system, which set temperatures ranging from 5 to 25 °C without shaking^52^, and the OD_600_ value was measured via a fluorescence spectrophotometer (TECAN, Switzerland). After selecting bacteria with relatively high growth rates at 13 °C, 1 × 10^6^ colony-forming units (CFU)/mL of each bacterium was inoculated in 5 mL of the R2A liquid. They were then cultured at 13 °C or 30 °C with 220 rpm of shaking for five days to find the psychrophilic bacterium with the best growth rate. The OD_600_ values of bacteria were measured over time (12 h unit intervals) using the spectrometer. *Escherichia coli* ATCC 25922 was used as a mesophilic bacterium for an experimental control.

### Identification of psychrophiles using 16S rRNA analysis

The genus of bacteria isolated from Antarctic soil was identified by partial sequencing of 16S rRNA genes. PCR amplification of the 16S rRNA gene was performed using ProFi Taq PCR PreMix (Bioneer, Oakland, USA). The PreMix PCR was used with 5 µL of RNase-free distilled water and 2 µL of each universal primer (27 F, 5′ AGAGTTTGATCMTGGCTCAG-3′, and 1492 R, 5′-GGTTACCTTGTTACGACTT-3′). The following PCR protocol was used: 95 °C for 5 min (1 cycle); 95 °C for 15 s, 58 °C for 15 s, 72 °C for 1 min (40 cycles), and 72 °C for 7 min (1 cycle). Pyrosequencing of the PCR product was performed by Macrogen (Republic of Korea). The obtained sequences were compared and classified using the EzTaxon Database (http://www.ezbiocloud.net).

### DNA extraction and total bacterial abundance

To assess variations in biomass (DNA) and cell density over a 28-day incubation period at temperatures of 13 °C and 30 °C, equal quantities of Antarctic soil samples (1 g) were introduced into R2A liquid medium. The enriched soil samples (10 mL) in the R2A medium diluted to 10^–1^ were collected on 0, 7, 14, and 28 days. The collected culture sample was centrifuged at 7800 rpm for 10 min, and the supernatant was filtered through 0.22 μm membrane filters (Sartorius, France). The filters with bacteria were then mixed with the collected bacteria and a soil pellet with 1 mL of distilled water. DNA extraction was performed using the FastDNA Spin Kit for soil (M.P. Biomedicals, USA). The concentration of the extracted DNA was measured using a Nanodrop 2000 spectrophotometer (Thermo Fisher Scientific, USA). The concentration of the DNA extracted from Antarctic soil was 10.4–515.5 ng/μL. Each DNA was used to analyze bacterial community analyses via 16S rRNA Nanopore sequencing and terminal restriction fragment length polymorphism (T-RFLP). Measuring CFU was performed to confirm the change in the total bacterial abundance from the Antarctic soil cultured for specific periods (0, 7, 14, and 28 days) in the R2A liquid medium with cycloheximide (100 µg/mL). The culture sample was serially diluted for measuring CFU to 10^–4^ using PBS after 7 days. Next, 100 μL of each dilution was streaked onto the R2A agar plate diluted to 10^–1^. The agar plates were incubated at 13 °C or 30 °C for 7 days. Colonies that appeared on the agar plates were counted to quantify total bacterial abundance.

### Analysis of bacterial communities in Antarctica soil using Nanopore sequencing tools

The DNA extraction was performed as described above. Bacterial community analyses of Antarctic soil samples were performed using Oxford Nanopore sequencing (Oxford Nanopore Technologies, U.K.). PCR reactions were performed using 10 ng of DNA from each sample, in addition to 25 µL of Long Amp Hot Start Taq 2X Master Mix and a 16S-specific barcoded primer pair (27F; AGA GTT TGA TCC TGG CTC AG, 1492R; GGT TAC CTT GTT ACG ACT T). The DNA samples were then purified using a 16S barcoding kit (SQK-16S024). DNA (100 ng/µL) was obtained by pooling equal concentrations of DNA for each sample after this sequencing was performed. The sequencing procedures rendered read counts of approximately Gigabases (Gb) using a single flow cell on a MinION portable sequencer (Oxford Nanopore Technologies, U.K.). Adapter trimming and assembly of raw sequences were performed using the CLC Genomic Workbench software version 20 (Qiagen, Germany). All samples underwent more than 200,000 reads, excluding the 0-day sample from Antarctic soil (111,021 reads), and the average sequence length was 203,427 (Table S7). Average reads passed the initial filter and were processed for taxonomic analysis with at least 75% identity. Alpha diversity and PCA analyses were also performed with the CLC Genomics Workbench software. Raw sequences for each sample were deposited in the NCBI BioSample under the accession numbers SAMN18043603 (13 °C_0 day), SAMN18043604 (13 °C_7 days), SAMN18043605 (13 °C_14 days), and SAMN18043606 (13 °C_28 days).

### Analysis of bacterial communities in Antarctica soil using T-RFLP

PCR amplification was performed using the PreMix PCR with 6-carboxyfluorescein labeled FAM_27F primer (5′-AGAGTTTGATCMTGGCTCAG-3′) and non-fluorescence labeled BAC519R primer (5′-GWATTACCGCGGCKGCTG-3′). The PCR cycle was set to 95 °C for 3 min (1 cycle); 94 °C for 30 s, 52.5 °C for 30 s, 72 °C for 90 s (35 cycles), and 72 °C for 10 min (1 cycle). The fluorescently labeled PCR products were digested with *MspI* or *Rsal* (Thermo Fisher Scientific, USA) for three hours at 37 °C, and then inactivation of the restriction enzyme was conducted for 15 min at 65 °C. The T-RFLP peaks were measured using an ABI PRISM 3730XL Analyzer (Applied Biosystems, USA), and the probable profiles of bacteria were identified for the EzTaxon Database. Peak values were aligned using the Peak scanner software version 1.0 (http://www.appliedbiosystems.com).

### *P. psychrotolerans* YJ56 growth under different temperature conditions

Psychrophilic *P. psychrotolerans* YJ56 (available as KACC 21510^T^, JCM 33881^T^) isolated from the Antarctic soil was cultured in R2A liquid medium at 13 °C with 220 rpm of shaking until the exponential phase (OD_600_:1.0). In accordance with the aim of the experiments, the *P. psychrotolerans* strain YJ56 was cultured in an R2A liquid medium at 13 °C and 25 °C until each exponential phase (OD_600_:1.0). Aggregation rates were quantified using a simple mild centrifuge (2,000 rpm) for 2 min and then the OD_600_ in the supernatant was measured for the non-aggregated portion. Aggregation rate was calculated as follows: Aggregation rate (%) = 1 − [(final OD_600_)/(initial OD_600_)].

### Observation of cellular morphology using microscopy

Strain YJ56 was observed by using Quanta 250 FEG field-emission SEM (SEM; JSM-6701F, JEOL, Japan), stereomicroscope (SZ61/SZ51, Olympus, Japan), phase-contrast, inverted microscopy (Carl Zeiss, USA), and confocal laser scanning microscopy (CLSM; Carl Zeiss, LSM700, USA). In the phase-contrast microscopy and SEM experiments, strain YJ56 was incubated in different media conditions until the exponential phase (OD_600_: 1.0) prior to microscopic observation. The phase-contrast microscopy observation was performed at 1000 × total magnification by placing 10 μL of the sample onto a glass slide. For the SEM observation according to the previous study, the cells were fixed with a low-strength Karnovsky Solution (2% paraformaldehyde, 2.5% glutaraldehyde, and 0.1 M phosphate buffer, with a final pH of 7.2) for 2 h, and further fixed with a 2% osmium tetroxide solution for 2 h^53^. The fixed samples were gradually dehydrated with different ethanol concentrations (30, 50, 70, and 100%) for 10 min at each gradient and placed onto an aluminium stub for 4 days to completely dry. The samples were coated with platinum and analyzed using the SEM. In the stereo- and inverted-microscopy experiments (25 × total magnification), strain YJ56 was incubated in 0.3% agar plates under different conditions for 14 days before microscopic observation. Cellular length and width, along with the colonies’ diameter, were measured using UVP Colony Doc-It Imaging Station (Analytik Jena, Germany) based on the SEM and stereomicroscope (4 × total magnification) images respectively. The inverted microscopy was used by adjusting to 25 × total magnification.

### Reverse transcription quantitative–PCR (qRT–PCR)

Total RNA extracted from the YJ56 strain, cultured at both 13 °C and 25 °C until reaching the exponential phase (with OD_600:_ 1.0) in R2A liquid (5 mL), was utilized for the qRT-PCR assay^54^. The RNA extraction was performed using the RNeasy Mini Kit (Qiagen, Germany). Using 1 μg of the total extracted RNA for each sample, cDNA was synthesized and subsequently amplified using the primers (RT-qPCR primers) listed in Table S8. The relative expression levels of individual genes were standardized against the 16S rDNA gene and evaluated employing the QuantStudio 3 Real-Time PCR instrument (Thermo Fisher Scientific, USA). The qRT-PCR protocols were executed in triplicate across a minimum of three distinct cultures. Statistical evaluations were carried out using a two-tailed Student’s *T*-test for pairwise comparisons between two groups (*p < 0.05, **p < 0.01, ***p < 0.001).

### Comparative genomic analysis and identification of insertion sequences

The complete sequences of strain YJ56 (NZ_CP047898.1) and five other *Pseudarthrobacter* species [*P. chlorophenolicus* A6 (GCA_000022025.1), *P. equi* IMMIB L-1606 (GCA_900105535.1), *P. phenanthrenivorans* Sphe3 (GCA_000189535.1), *P. siccitolerans* 4J27 (GCA_001046895.1), and *P. sulfonivorans* Ar51 (GCA_001484605.1)] were retrieved from the Integrated Microbial Genomes (IMG) platform. Comparative genome analysis was conducted using Mauve software (http://darlinglab.org/mauve/download.html). The complete genomic sequence and homology of the *rimJ* gene in strain YJ56 were analyzed using the CLgenomics program version 1.55 (ChunLab, South Korea) and the UniProt database (https://www.uniprot.org/blast/). The GC ratio was measured with the GC Content Calculator (https://www.biologicscorp.com/tools/GCContent/#.XnIhjqgzZPY). Orthologous average nucleotide identity (OrthoANI) and OrthoVenn2 (https://orthovenn2.bioinfotoolkits.net) analyses were conducted to compare genetic relatedness and orthologous clusters with six other *Pseudarthrobacte*r species^55–57^. The web tool OrthoVenn with E-value (1e−2) was used to compare and annotate the clusters of orthologous groups, and to perform a Gene Ontology (GO) enrichment analysis. Insertion sequence (IS) elements of genomic sequences in the *Pseudarthrobacter* species were analyzed with ISfinder (https://isfinder.biotoul.fr/blast.php). Insertion sequences with *E*-value ≥ 0.0001 were counted. Phylogenetic trees based on the 16S rRNA or *rimJ* gene [encoding (ribosomal protein S5)-alanine *N*-acetyltransferase] sequences were constructed using maximum-likelihood algorithms with MEGA version 7.0 (http://www.megasoftware.net); a bootstrap test was evaluated with 1000 replicates. The evolutionary distances were calculated using the maximum composite likelihood method^57–59^. The obtained 16S rRNA sequences were compared and classified using the EzBioCloud database (http://www.ezbiocloud.net). The *rimJ* gene sequences in the complete genomic sequence of strain YJ56 were obtained using CLgenomics version 1.55. These gene sequences were aligned using MEGA software; a bootstrap test was evaluated with 1000 replicates. The complete genomic sequence of each bacterium was analyzed using the EzBioCloud database to identify the copy number of *rimJ*, *rimI*, and *rimL* genes.

### Two-dimensional gel electrophoresis (2-DE) and image analyses

Strain YJ56 was cultured in an R2A liquid medium at 13 °C or 25 °C until the exponential phase (OD_600_: 1.0) with 220 rpm. To obtain cell lysates for 2-DE analysis, cell pellets were treated with a protease inhibitor complete cocktail tablet (Roche, Germany) and a sample buffer (7 M urea, 2 M thiourea, 4.5% (w/v) 3-[(3-cholamidopropyl) dimethylammonio]-1-propanesulfonate (CHAPS), 100 mM dithioerythritol (DTE), 40 mM Tris, pH 8.8). The samples were then sonicated for 20 s with 75% amp by using a Sonopuls sonicator (Bandelin, Germany). After centrifugation at 15,000×*g* for 1 h at 15 °C, the insoluble material was discarded and the soluble fraction was used for the 2-DE analysis. Protein loading was normalized by measuring protein concentrations with the Bradford assay. The lysates were applied to immobilized pH 3–10 nonlinear gradient strips (Amersham Biosciences, U.K.). Isoelectric focusing (IEF) was performed at 80,000 Vt. The second dimension was analyzed on 9–16% linear gradient polyacrylamide gels (18 cm × 20 cm × 1.5 mm) at a constant 40 mA per gel for approximately 5 h. After protein fixation in a solution of 40% methanol and 5% phosphoric acid for 1 h, the gels were stained with Coomassie brilliant blue (CBB) G-250 for 12 h. The gels were destained with distilled water, scanned in a Bio-Rad GS710 densitometer (Richmond, USA), converted into electronic files, and then analyzed with Image Master Platinum 5.0’s image analysis program (Amersham Biosciences). The quantity of protein in each spot was normalized to the total valid spot intensity. By comparing each gel image of proteins in strain YJ56 under different temperature conditions (13 °C or 25 °C), each spot’s quantity was normalized against the total valid spot intensity. Proteins with an increased abundance of more than 2.0-fold at 13 °C were identified in 21 of the total 332 spots (Table S9). The identified 37 of the 409 total spots at 25 °C were indicated as protein expressed more than 2.0-fold (Table S10). According to temperature conditions, the numbers of only observed spots were 94 and 171 spots at 13 °C and 25 °C, respectively (Table S11, Table S12). Protein spots were finally selected when the variation in intensity increased/decreased more than 3.0-fold or had more than 0.5 volume value at each temperature. Also, spots with a volume ratio above 0.1% were chosen among the spots uniquely identified under each temperature condition.

### Liquid chromatography-mass spectrometry (LC–MS/MS) for peptides analysis and database searching

Nano LC–MS/MS analysis was performed with an Easy-nLC and an LTQ Orbitrap XL mass spectrometer equipped with a Nano-electrospray source (Thermo Fisher, San Jose, CA, USA). Samples were separated on the C18 Nanobore column (150 mm × 0.1 mm, 3 μm of pore size; Agilent). Mobile phase A for L.C. separation was 0.1% formic acid and 3% acetonitrile in deionized water. Mobile phase B was 0.1% formic acid in acetonitrile. The chromatography gradient was designed for a linear increase from 0% B to 60% B in 9 min, 60% B to 90% B in 1 min, and 3% B in 5 min. The flow rate was maintained at 1800 nL/min. Mass spectra were collected using data-dependent acquisition with a whole mass scan (380–1700 m*/z*) followed by 10 MS/MS scans. For MS1 full scans, the Orbitrap resolution was 15,000, and the AGC was 2 × 10^5^. For MS/MS in the LTQ, the AGC was 1 × 10^4^. The Mascot Algorithm (Matrix Science, USA) was used to identify peptide sequences present in a protein sequence database. Database search criteria were taxonomy; *P. psychrotolereans* YJ56 (4227 sequences; 1,310,136 residues), and *P. sulfonivorans* ALL (8781 sequences; 2,858,961 residues) as phylogenetically related species, fixed modification; carbamidomethylation at cysteine residues; variable modification; oxidized at methionine residues, maximum allowed missed cleavage; 2, M.S. tolerance; 10 ppm, MS/MS tolerance; 0.8 Da. The peptides were filtered with a significance threshold of *P* < 0.05. The LC–MS/MS-based proteomics data of all identified peptides and protein lists have been deposited in the ProteomeXchange Consortium http://proteomecentral.proteomexchange.org) via the PRIDE partner repository (accession number, PXD040138).

### Fatty acid methyl esters (FAME)

Strain YJ56 was cultured in an R2A liquid medium at 13 °C or 25 °C until the stationary phase (OD_600_: ~ 3.0), with 220 rpm of shaking before FAME analysis. The preparation and extraction of the fatty acid samples were performed by standard methods. Bacterial cells were harvested by centrifugation and were washed with PBS before being resuspended in 1 mL of solution I (Saponification reagent; 7.5 g of sodium hydroxide, 25 mL of distilled water, and 25 mL of methanol for 50 mL of total volume). The bacterial samples were vortexed for 30 s, heated at 100 °C for 5 min, vortexed for another 10 s, and heated once again at 100 °C for 25 min. The heated samples were cooled at ambient temperature for 1 min and then treated to 2 mL of solution II (Methylation reagent; 27.1 mL of 6N HCl and 22.9 mL of methanol for 50 ml of total volume). Next, the samples were vortexed for 10 s and heated at 80 °C for 10 min. 1.25 mL of solution III (Extraction solvent; 25 mL of hexane and 25 mL of methyl-tert butyl ether for 50 mL of total volume) was added to the samples after cooling at ambient temperature for 1 min. The samples were given 3 mL solution IV (Base wash reagent; 0.6 g sodium hydroxide and 50 mL distilled water for 50 mL of total volume) and vortexed for 5 min after the samples’ upper layer was transferred into a new 15 mL-conical tube. Finally, the upper layer of samples was transferred into G.C. vials. The MIDI/Hewlett Packard Microbial Identification System (Agilent 7890a G.C., USA) was used to investigate FAME. The washing solvent was 99.9% hexane, and the temperature program of the GC-MIDI was set (Oven, 300; Front Inlet, 280; Front Detector, 310 °C). The flame ionization detector allowed for an extensive dynamic range and provided good sensitivity. Hydrogen was the carrier gas, nitrogen was the “makeup” gas, and the air was used for supporting the flame.

### Supplementary Information

Supplementary Information.

## Data Availability

The datasets generated during the current study are available in the NCBI sequence archives under accession numbers NZ_CP047898.1 (*P. psychrotolerans* YJ56), GCA_000022025.1 (*P. chlorophenolicus* A6), GCA_900105535.1 (*P. equi* IMMIB L-1606), GCA_000189535.1 (*P. phenanthrenivorans* Sphe3), GCA_001046895.1 (*P. siccitolerans* 4J27), GCA_001484605.1 (*P. sulfonivorans* Ar51), and NC_000913.3 (*E. coli* MG1655). The remaining datasets generated during the current study are available in the NCBI BioSample under accession numbers SAMN18043603 (13 °C_0 day), SAMN18043604 (13 °C_7 days), SAMN18043605 (13 °C_14 days), and SAMN18043606 (13℃_28 days). The proteomic data of all identified peptides and protein lists have been deposited in the ProteomeXchange Consortium (http://proteomecentral.proteomexchange.org) via the PRIDE partner repository, with the data set identifier PXD040138 (proteomics analyses of strain YJ56 cultured at 13 °C and 25 °C_proteomic data).
