# Supplementary material for: Morphological and physiological adaptations of psychrophilic Pseudarthrobacter psychrotolerans YJ56 under temperature stress
Source: Sci Rep. 2023 Sep 11;13:14970. doi: 10.1038/s41598-023-42179-x (PMC10495460; doi:10.1038/s41598-023-42179-x)

Morphological and physiological adaptations of psychrophilic *Pseudarthrobacter psychrotolerans* YJ56 under temperature stress

Yongjun Son[1], Jihyeon Min[1], Yoonjae Shin[1], Woojun Park[1*]

[1]Laboratory of Molecular Environmental Microbiology, Department of Environmental Science and Ecological Engineering, Korea University, Seoul 02841, Republic of Korea

Keywords: Antarctic; extremophile; bacterial community; cell elongation; branched filaments; oligotrophic

[*]**Corresponding author:** Dr. Woojun Park, Department of Environmental Science and Ecological Engineering, Korea University, Seoul 02841, Republic of Korea

E-mail: wpark@korea.ac.kr

Fax: +82-2-953-0737

Phone: +82-2-3290-3067

Table S1. Identification of the genus isolated from Antarctic soil based on 16S rRNA sequences.

Source	Culture medium	Sample no. (Total: 39)	16S rRNA analysis
Antarctic soil	R2A	2, 6, 7, 8, 9, 11, 18, 21, 23, 26, 28, 30, 32, 33, 35, 38, 39, 40, 42, 54, 56	*Pseudarthrobacter*
		1, 4, 15, 17, 22, 34, 53, 60	*Arthrobacter*
		12, 13, 16, 43, 50, 52	*Rhodococcus*
		3, 25, 37	*Leifsonia*
		20	*Sporosarcina*

Table S2. General genome features of *P. psychrotolerans* YJ56 and five species closest to *Pseudarthrobacter* based on OrthoANI analysis.

	**Value for *Pseudarthrobacter* species**					
**Parameter**	***P. psychrotolerans* YJ56**	*P. sulfonivorans* Ar51	*P. siccitolerans* 4J27	*P. phenanthrenivorans* Sphe3	*P. chlorophenolicus* A6	*P. equi* IMMIB L-1606
Genome size (Mp)	**5.17**	4.39	4.77	4.55	4.40	4.46
GC content (%)	**64.7**	65.1	65.1	65.3	66.3	66.1
No. of plasmid	**2**	1	0	1	2	0
No. of tRNA	**51**	50	52	50	49	50
No. of rRNA	**15**	3	5	3	15	15
No. of genes	**4907**	4638	4443	4288	4715	4118
No. of protein-coding sequences	**4838**	4257	4384	4212	4561	3968

Habitat	**Antartica soil**	Permafrost	*Nerium oleander* rhizosphere	Creosote contaminated soil	Soil slurry	Genital swabs of a horse
Optimal growth temperature (°C)	**13**	28	30	28	28	28

Table S3. Unique proteins in *P. psychrotolerans* YJ56 were determined using BLASTP on the NCBI database. Proteins are categorized by related function. Hypothetical proteins were not listed.

Protein name	Species with the closest sequence	Accession number	Query/Template length (aa)	Homology (%)
**Transcriptional regulator (n: 18)**				
DEAD/DEAH box helicase family protein	*Arthrobacter* sp. 31Y	WP_024821119.1	534/711	75.11
DEAD/DEAH box helicase family protein	*Arthrobacter* sp. 31Y	WP_024821119.1	1288/1786	72.12
DEAD/DEAH box helicase family protein	*Arthrobacter* sp. 31Y	WP_024821119.1	390/673	57.95
DEAD/DEAH box helicase family protein	*Arthrobacter* sp. 31Y	WP_024821119.1	783/969	80.80
DEAD/DEAH box helicase family protein	*Arthrobacter* sp. 31Y	WP_024821119.1	616/806	76.43
IclR family transcriptional regulator	*Arthrobacter* sp. 4R501	WP_104063627.1	156/184	97.28
IclR family transcriptional regulator	*Pseudarthrobacter* sp. NIBRBAC000502772	QDG68393.1	236/254	94.02
IclR family transcriptional regulator	*Arthrobacter* sp. AQ5-06	WP_111909391.1	234/244	95.90
IclR family transcriptional regulator	*Pseudarthrobacter* sp. NBSH8	WP_185261791.1	243/247	98.38
Metallo-regulator ArsR/SmtB family transcription factor	*Arthrobacter stackebrandtii*	WP_209682438.1	76/115	66.09
PucR family transcriptional regulator	*Arthrobacter* sp. Soil762	WP_056339627.1	334/396	84.34
RNA polymerase subunit sigma factor 70	*Arthrobacter* sp. 31Y	WP_051483152.1	172/179	83.15
RNA polymerase subunit sigma factor 70	*Pseudarthrobacter* sp. AB1	MBE4720478.1	182/198	91.92
RNA polymerase subunit sigma factor 70	*Arthrobacter* sp. SRS-W-1-2016	WP_171983506.1	263/265	99.25
Single-stranded DNA-binding protein	*Arthrobacter* sp. 31Y	WP_024821121.1	107/155	69.00

Single-stranded DNA-binding protein	*Arthrobacter* sp. 31Y	WP_024821121.1	100/180	55.56
TetR family transcriptional regulator	*Streptomyces acidiscabies*	WP_050375701.1	121/183	66.12
TetR family transcriptional regulator	*Arthrobacter* sp.	HAP91264.1	218/227	96.04
**Mobile genetic element (n: 13)**				
IS1380 family transposase	*Arthrobacter* sp. Y81	WP_104174753.1	441/464	95.04
IS1380 family transposase	*Arthrobacter* sp. AQ5-06	WP_111909753.1	57/69	82.61
IS701 family transposase	*Arthrobacter* sp. Soil736	WP_056626690.1	353/365	97.26
IS701 family transposase	*Arthrobacter oryzae*	WP_123253660.1	347/377	92.04
IS701 family transposase	*Arthrobacter* sp. 24S4-2	WP_137321512.1	348/365	96.44
IS701 family transposase	*Arthrobacter* sp. Soil736	WP_056626690.1	354/365	96.99
Site-specific integrase	*Paenarthrobacter* sp. AR 02	WP_235516950.1	256/363	70.82
Site-specific integrase	*Pseudarthrobacter* sp. AB1	WP_193339967.1	303/306	99.02
Site-specific recombinase XerD	*Arthrobacter* sp. ok909	SDP65015.1	445/504	88.29
Site-specific recombinase XerD	*Arthrobacter* sp. ok909	SDP65015.1	461/515	89.51
Tn3 family transposase	*Arthrobacter psychrochitiniphilus*	WP_110106784.1	883/988	89.37
Tn3 family transposase	*Propionivibrio* sp.	MBP7525526.1	586/986	59.43
Transposase	*Rhodococcus* sp. WS4	TQC39056.1	77/122	63.11
**Transporter (n: 10)**				
ABC transporter substrate-binding protein	*Arthrobacter* sp. 31Y	HAG59096.1	300/302	99.34

ABC transporter substrate-binding protein	*Arthrobacter* sp. 31Y	HAG58416.1	284/308	92.21
Amino acid ABC transporter permease	*Arthrobacter* sp. AQ5-06	WP_111908091.1	285/307	92.83
Amino acid ABC transporter permease	*Microbacteriaceae bacterium*	WP_160669473.1	160/226	70.80
Amino acid ABC transporter permease	*Arthrobacter* sp. 24S4-2	WP_137323630.1	287/317	90.54
Amino acid ABC transporter permease	*Pseudarthrobacter* sp. SSS035	WP_248758372.1	218/223	97.76
MFS transporter	*Pseudarthrobacter* sp. AB1	WP_193341637.1	449/463	96.98
MFS transporter	*Arthrobacter* sp. Soil762	WP_056340999.1	436/452	96.46
PhnD/SsuA/transferrin family substrate-binding protein	*Arthrobacter* sp. AQ5-06	WP_111907981.1	262/270	97.04
Transporter substrate-binding domain-containing protein	*Pseudarthrobacter* sp. SSS035	WP_248758374.1	271/279	97.13
**N-acyltransferase (n: 5)**				
GNAT family N-acetyltransferase	*Micrococcaceae*	WP_069952983.1	168/168	100
GNAT family N-acetyltransferase	*Arthrobacter* sp. AQ5-06	WP_111909334.1	154/156	98.17
GNAT family N-acetyltransferase	*Arthrobacter* echini	WP_148601822.1	126/153	82.81
GNAT family N-acetyltransferase	*Arthrobacter* sp. QL17	WP_069952983.1	133/167	79.64
GNAT family N-acetyltransferase	*Arthrobacter* sp. Br18	WP_051476674.1	121/164	73.78
**Hydrolase activity (n: 10)**				
Amidohydrolase	*Arthrobacter* sp. 24S4-2	WP_137322178.1	536/557	93.00
Amidohydrolase family protein	*Arthrobacter* sp. PO-11	MBO1267589.1	435/488	89.17

Amidohydrolase family protein	*Pseudarthrobacter* sp. NBSH8	WP_185261684.1	476/494	96.36
Glycoside hydrolase family 15 protein	*Arthrobacter* sp. AQ5-06	WP_111907979.1	577/615	93.82
Glycoside hydrolase family 15 protein	*Arthrobacter* sp. U41	AOT05423.1	558/613	91.30
Rid family hydrolase	*Arthrobacter* sp. AQ5-06	WP_111909389.1	125/126	99.32
Rid family hydrolase	*Pseudarthrobacter* sp. NIBRBAC000502772	WP_246848620.1	251/278	90.90
Rid family hydrolase	*Arthrobacter* sp. AQ5-06	WP_111909324.1	129/134	96.27
RidA family hydrolase	*Plantibacter* sp. VKM Ac-2876	WP_194599892.1	110/130	84.62
RidA family hydrolase	*Pseudarthrobacter* sp. NIBRBAC000502772	WP_141141666.1	116/119	97.48
**Toxin-antitoxin system (n: 7)**				
Nucleotidyl transferase AbiEii/AbiGii toxin family protein	*Arthrobacter ramosus*	WP_234750667.1	169/213	79.40
Type II toxin-antitoxin system HipA family toxin	*Arthrobacter* sp. AQ5-06	WP_111909087.1	93/116	80.17
Type II toxin-antitoxin system HipA family toxin	*Arthrobacter silviterrae*	WP_165181828.1	340/460	73.91
Type II toxin-antitoxin system PemK/MazF family toxin	*Arthrobacter* sp. FW305-BF8	WP_236809045.1	91/100	91.00
Type II toxin-antitoxin system PemK/MazF family toxin	*Arthrobacter* sp. ISL-72	WP_214841235.1	96/108	88.89
Type II toxin-antitoxin system Phd/YefM family antitoxin	*Arthrobacter* sp. SLBN-112	WP_142031450.1	74/81	91.36
Type II toxin-antitoxin system Phd/YefM family antitoxin	*Arthrobacter* sp. SLBN-112	WP_142031450.1	73/81	90.12
**Secretion system**				

Type VII secretion protein EccE	*Arthrobacter* sp. U41	WP_083266778.1	545/549	99.27
Type VII secretion protein EccE	*Arthrobacter* sp. U41	WP_083266778.1	544/549	99.00
**Stress response (n: 1)**				
CsbD family protein	*Arthrobacter* sp. 4R501	WP_056340807.1	57/58	98.28

Table S4. Insertion sequence analysis of the "A" site with abundant *rimJ* copies in genomic sequence of strain YJ56 (114,355 to 121,867 bp).

IS Family	Group	Accession number	Origin	Host	GC ratio (%)	Score (bits)	E-value
IS5	IS427	X65618	*Mycobacterium tuberculosis*	*Mycobacterium tuberculosis* H37Rv<br>*Mycobacterium tuberculosis* Johnston<br>*Mycobacterium bovis* BCG	64.0	67.9	2e-10
IS5	IS427	AL079356	*Streptomyces coelicolor*	*Streptomyces coelicolor* cosmid 6G9	66.0	60.0	5e-08
IS5	IS427	X65618	*Azospirillum* sp.	*Azospirillum* sp. B510 plasmid pAB510b<br>*Azospirillum* sp. B510 plasmid pAB510a<br>*Azospirillum* sp. B510<br>*Azospirillum* sp. B510 plasmid pAB510d<br>*Azospirillum* sp. B510 plasmid pAB510c	59.0	48.1	2e-04
IS5	IS427	AL079356	*Rhizobium etli*	*Rhizobium etli* CFN 42 symbiotic plasmid p42d<br>*Rhizobium etli* Kim 5	58.0	48.1	2e-04

Table S5. Identification of proteins expressed from strain YJ56 cultured at two different temperatures.

Accession number[a]	SSP	Gene	Protein	Change[b] (fold)	N-terminal sequence	Theoretical		Experimental		Sequence coverage (%)
						Mr (KDa)	pI	Mr (KDa)	pI	
**Proteins with increased abundance at 13 °C compared to 25 °C**										
QHK20964.1	39	*parB*	Chromosome partition protein ParB	2.7	ISLGVRK	45.7	5.3	75.0	4.3	1
QHK19950.1	188	*tufA*	Elongation factor Tu	6.6	TTLTAAISK	43.6	5.1	43.0	5.1	33
QHK20979.1	338	*cheB2*	Response regulator	5.7	AITASGAGA R	25.0	4.8	30.0	5.2	4
QHK19930.1	566	*rplR*	L18 in 50S ribosomal protein	3.0	IAAVADGAR EGGLSL	13.7	11.5	14.0	3.8	47
QHK19939.1	601	*rpmC*	L29 in 50S ribosomal protein	3.7	KAESAETVE AEEDAK	12.2	6.6	12.0	6.6	84
QHK22541.1	763	hypothetical gene	Methyltransferase domain-containing protein	2.3	GVDSASASI DRAR	29.4	4.7	31.0	5.9	4
**Proteins only identified at 13 °C**										
QHK19573.1	22	hypothetical gene	DUF21 domain-containing protein		DATQATGIR	38.4	4.7	95.0	4.3	2
QHK21012.1	23	*hppD*	TIM barrel domain		LAALAVDH GVK	69.9	5	95.0	4.3	1
QHK19950.1	183	*tufA*	Elongation factor Tu		TKPHVNIGTI GHVDHGK	43.6	5.1	43.0	4.6	44
QHK19900.1	227	*YycA*	Phospholipid carrier-dependent glycosyltransferas		ATNVQALG GTPLKR	66.2	9.2	41.0	5.8	2
**Proteins with increased abundance at 25 °C compared to 13 °C**										

QHK22130.1	56	*infC*	Translation initiation factor IF-3	3.8	RASQRAEAK	39.4	9.6	68.0	4.8	2
QHK21642.1	93	*groEL*	Chaperone GroEL	7.0	TDDVAGDG TTTATVLAQ ALVK	56.7	4.8	57.0	4.9	64
gi|971342525	109	*groEL*	Chaperone GroEL	3.0	DNTTIVDGA GSAEDVAAR	56.4	4.9	51.0	5.1	42
QHK19950.1	182	*tufA*	Elongation factor Tu	5.4	TKPHVNIGTI GHVDHGK	43.6	5.1	44.0	5.2	38
QHK21642.1	251	*groEL*	Chaperone GroEL	2.1	NVAAGADPL SLKR	56.7	4.8	35.0	5.0	32
QHK22188.1	312	hypothetical gene	Four-helix bundle copper-binding protein	5.3	TVLEAFRR	11.8	4.6	30.0	4.6	7
QHK18875.1	328	*seA*	Sulfurtransferase	4.4	DTTVVIYGD K	33.3	4.8	31.0	4.8	31
QHK21606.1	382	*gpmA*	Phosphoglyceromutase	6.4	RAINTANISL DKADR	27.8	5.0	28.0	5.1	31
QHK20003.1	459	hypothetical gene	NAD(P)H-binding protein	3.6	SMDAAAEA GVGR	22.1	4.8	22.0	4.8	42
QHK19948.1	619	*rpsJ*	S10 in 30S ribosomal protein	2.6	AGATVVGPV PLPTEK	11.7	9.4	11.0	4.4	63
**Proteins only identified at 25 °C**										
QHK21106.1	47	hypothetical gene	Amidohydrolase family protein		EEPAFALTR	51.6	5	72.0	5.2	1
gi|971342321	86	*katE*	Catalase KatE		YHFKSNQGV K	54.6	5.4	58.0	5.5	2
QHK19486.1	105	*ilvD*	Dihydroxy-acid dehydratase		SAGFDADVF EGTAR	60.5	5.3	50.0	5.3	39

gi|971342525	110	*groEL*	Chaperone GroEL	DNTTIVDGA GSAEDVAAR	56.4	4.9	51.0	5.4	57
QHK22089.1	145	*glyS1*	Glycine--tRNA ligase	YTPYVIEPA AGLTR	52.8	5.2	49.0	5.3	49
QHK18837.1	154	*pepA*	Leucyl aminopeptidase	VLGVTGAA DQIVR	52.2	6.0	49.0	6.2	34
QHK18732.1	177	*argG*	Argininosuccinate synthase	FEVGIQTLGP DLK	43.8	5.1	44.0	5.1	38
QHK19247.1	445	*rpsD*	S4 in 30S ribosomal protein	ALGIALTPK	23.8	10.9	24.0	5.1	48

a. Protein accession numbers from *Pseudarthrobacter psychrotolerans* YJ56 or *P. sulfonivorans* Ar51.

b. Changes in protein levels are reported as the ratio between the normalized protein spot volume from cells grown at 13 °C and 25°C ($V_{13\ °C}/V_{25\ °C}$ or $V_{25\ °C}/V_{13\ °C}$, %), for proteins present at a higher level.

Table S6. Comparison of the cellular fatty acid compositions (%) of strain YJ56 cultured in an R2A liquid medium at 13 ℃ or 25 °C. The data represents percentages of the total fatty acids. Highlighted percentages in bold indicate major fatty acid components (>5.0%). –, Not detected.

Fatty acid	13 °C	25 °C
**Saturated straight chain:**		
$C_{9:0}$	0.8	0.5
$C_{14:0}$	2.1	1.2
$C_{16:0}$	2.1	5.9
$C_{18:0}$	0.3	0.6
**Saturated branched:**		
iso-$C_{14:0}$	1.6	0.7
iso-$C_{15:0}$	2.3	10.1
anteiso-$C_{15:0}$	51.9	53.1
anteiso-$C_{15:0}$ A	1.9	–
iso-$C_{16:0}$	2.3	3.8
iso-$C_{16:1}$ H	1.3	0.3
anteiso-$C_{17:0}$	3.5	13.6
**Unsaturated:**		
$C_{14:1}\omega5c$	3.4	1.3
anteiso-$C_{17:1}\omega9c$	3.6	1.4
**Hydroxy:**		
$C_{8:0}$ 3-OH	2.1	1.2
iso-$C_{17:0}$ 3-OH	2.3	0.8
**Summed feature*:**		
3	11.8	2.1

*Summed features represent groups of two or three fatty acids that could not be separated by GLC with the MIDI system. Summed feature 3 contains $C_{16:1}\omega7c$ and/or $C_{16:1}\omega6c$.

Table S7. Statistics of Nanopore sequencing (MinION) data in Antarctic soil.

Antarctica Soil (13℃)	Reads length (bp)				
	Total reads	Min	Median	Max	Mean
0 day	111,021	136	1,542	4,715	1,460
7 days	248,963	140	1,549	4,685	1,387
14 days	219,306	122	1,550	4,859	1,406
28 days	234,185	112	1,544	4,728	1,337

Table S8. Primers were used in this study.

Gene	Primer sequence (5`-3`)	Reference
16S rRNA_F	CGCAAGCGTTATCCGGAAT	This study
16S rRNA_R	TTGAGCCCCGGACTTTCAC	This study
*ftsZ*_F	GCAGCTCCGCAGAATTACTTG	This study
*ftsZ*_R	CGAAGACCGACCTCGATCA	This study
*divIVA*_F	CGTTGTCAACAAGCGCTTTC	This study
*divIVA*_R	TCTCGTCCAGGAAGTCATCAACT	This study

Table S9. Proteins with an increased abundance in strain YJ56 cultured at 13 °C compared with 25 °C. The quantity of protein in each spot was normalized to the total valid spot intensity. Through comparing each protein gel image in strain YJ56 under different temperature conditions, significantly changed spots were selected based on a rate increased/decreased 1.8-fold or by complete appearance or disappearance. The final number of selected proteins for LC/MS analysis was 5 (bold) among 21 identified spots.

SpotID	13 °C (% vol)	25 °C (% vol)	13 °C /25 °C (% vol) ratio
**39**	**3.793**	**1.425**	**2.7**
49	0.375	0.184	2
57	0.031	0.013	2.3
72	0.621	0.305	2
76	0.239	0.097	2.5
101	0.145	0.045	3.3
119	0.122	0.061	2
152	0.18	0.085	2.1
**188**	**1.182**	**0.178**	**6.6**
198	0.057	0.027	2.2
199	0.395	0.153	2.6
213	0.315	0.143	2.2
232	0.251	0.074	3.4
280	0.068	0.03	2.2
**338**	**0.52**	**0.091**	**5.7**
372	0.492	0.215	2.3
436	0.072	0.035	2.1
536	0.311	0.155	2
**566**	**3.414**	**1.155**	**3**
**601**	**0.396**	**0.106**	**3.7**
763	0.104	0.046	2.3

Table S10. Proteins with an increased abundance in strain YJ56 cultured at 25 °C compared with 13 °C. The quantity of protein in each spot was normalized to the total valid spot intensity. Through comparing each protein gel image in strain YJ56 under different temperature conditions, significantly changed spots were selected based on a rate increased/decreased 1.8-fold or by complete appearance or disappearance. The final number of selected proteins for LC/MS analysis was 13 (bold) among 37 identified spots.

SpotID	25 °C (% vol)	13 °C (% vol)	25 °C /13 °C (% vol) ratio
34	0.05	0.011	4.4
42	0.222	0.052	4.3
**56**	**1.708**	**0.45**	**3.8**
75	0.106	0.053	2
**93**	**2.023**	**0.289**	**7**
**109**	**1.508**	**0.51**	**3**
122	0.054	0.025	2.1
146	0.081	0.028	2.9
156	0.069	0.029	2.4
**182**	**3.204**	**0.589**	**5.4**
187	0.162	0.073	2.2
206	0.235	0.056	4.2
237	0.117	0.059	2
246	0.096	0.028	3.4
**251**	**1.344**	**0.639**	**2.1**
281	0.741	0.251	3
298	0.454	0.119	3.8
**312**	**0.598**	**0.112**	**5.3**
**328**	**0.773**	**0.177**	**4.4**
335	0.155	0.035	4.4
340	0.293	0.125	2.3
346	0.492	0.216	2.3
366	0.09	0.033	2.7
**382**	**0.39**	**0.061**	**6.4**
389	0.143	0.055	2.6

437	0.307	0.138	2.2
438	0.453	0.146	3.1
446	0.128	0.065	2
453	0.616	0.148	4.1
**459**	**0.677**	**0.186**	**3.6**
468	0.205	0.05	4.1
500	0.191	0.079	2.4
510	0.237	0.117	2
526	0.225	0.107	2.1
564	0.169	0.074	2.3
**619**	**2.277**	**0.875**	**2.6**
751	0.059	0.029	2

Table S11. Proteins only identified in strain YJ56 cultured at 13 °C. Strain YJ56 expressed 94 proteins at 13 °C. The number of selected proteins for LC/MS analysis was 4 spots (bold).

SpotID	X	Y	Area	Vol	%Vol
9	393	334	15.7077	1318.69	0.115853
14	396	361	9.17908	879.406	0.07726
**22**	**375**	**502**	**15.3589**	**2348.27**	**0.206305**
**23**	**407**	**507**	**14.1174**	**2204.56**	**0.19368**
30	324	530	11.0484	778.869	0.068427
32	405	537	8.50948	822.748	0.072282
36	410	568	8.09098	910.048	0.079952
37	399	594	13.2525	1763.15	0.1549
39	165	610	29.3647	4375.77	0.38443
46	228	618	7.46323	772.089	0.067831
47	247	618	19.0557	2272.06	0.19961
52	285	633	21.0087	2969.86	0.260915
65	351	706	6.04034	704.906	0.061929
66	371	712	7.95148	1160.14	0.101923
67	391	713	8.46763	1471.53	0.12928
84	371	787	18.6511	2940.56	0.258341
95	412	800	21.9712	4404.91	0.38699
96	351	803	15.4705	1577.35	0.138577
102	1202	809	7.03079	325.732	0.028617
126	580	885	19.8648	3565.51	0.313246
132	1168	911	5.67764	270.704	0.023783
133	1200	915	8.11888	371.99	0.032681
140	1330	930	7.07264	701.124	0.061597
159	468	976	10.1137	1366.87	0.120086
**183**	**466**	**1077**	**66.7088**	**24037.2**	**2.11177**
201	2213	1085	10.4067	554.19	0.048688
213	577	1130	10.8391	1289.14	0.113257
219	369	1136	6.77969	900.652	0.079126
**227**	**1175**	**1154**	**8.85823**	**1001.62**	**0.100997**
229	279	1158	12.7921	1070.59	0.094056
232	318	1157	13.4059	2143.23	0.188292
234	541	1173	22.7524	4554.22	0.400108

256	950	1228	9.12328	373.921	0.032851
257	2192	1229	9.65338	274.005	0.024073
259	516	1234	11.6343	2236.31	0.196469
264	361	1250	11.5506	1800.97	0.158223
275	1313	1282	10.323	703.302	0.061788
276	1223	1281	9.83473	598.858	0.052612
278	1243	1284	6.20774	329.824	0.028976
280	665	1290	8.92798	783.026	0.068792
299	147	1343	14.8567	2281.94	0.200478
301	639	1348	15.345	2180.39	0.191557
305	537	1368	7.24003	938.694	0.082468
308	935	1377	10.5183	1139.84	0.10014
310	892	1382	10.6438	903.135	0.079344
317	496	1395	16.9911	2167.78	0.190449
321	264	1405	15.331	1419.49	0.124709
329	653	1419	7.77013	757.417	0.066542
357	128	1530	11.5506	1236.96	0.108672
363	258	1558	10.6299	977.084	0.085841
365	310	1558	8.87218	979.804	0.08608
366	356	1561	10.4346	1009.62	0.088699
373	141	1570	10.2951	1108.19	0.097359
389	1221	1615	8.36998	524.775	0.046104
394	905	1628	14.3964	1157.9	0.101727
398	246	1637	14.5359	621.185	0.054574
408	147	1659	29.2531	9672.9	0.849805
412	274	1675	27.3698	4818.39	0.423316
419	1222	1679	7.39348	559.994	0.049198
424	181	1686	36.6466	8165.07	0.717337
425	631	1689	15.9309	1566.33	0.137609
428	211	1708	17.3259	3581.77	0.314674
434	153	1721	10.2672	1184.92	0.104101
439	119	1732	11.7738	1556.93	0.136783
441	1687	1736	7.79803	411.203	0.036126
447	273	1773	35.6003	7132.97	0.626662
453	490	1770	16.8655	1472.5	0.129366

460	903	1815	12.7782	1165.97	0.102435
469	206	1873	25.4587	3373.21	0.296351
472	247	1874	21.4272	3407.92	0.299401
473	140	1880	24.803	3509.49	0.308324
478	1220	1901	13.8802	753.019	0.066156
510	149	2019	75.6925	31844.8	2.7977
521	1225	2049	12.0807	904.195	0.079437
561	1220	2129	9.49993	1181.41	0.103792
567	269	2150	28.0394	11545.8	1.01435
573	1226	2145	8.90008	570.946	0.05016
576	2316	2150	12.8479	1247.08	0.109561
577	576	2151	9.20698	1286.82	0.113052
585	2285	2166	13.8802	698.867	0.061399
629	2276	2239	17.8978	2486.33	0.218435
632	1453	2246	12.8758	1423.41	0.125053
649	1686	2261	9.66733	936.015	0.082233
652	1457	2269	11.16	1517.06	0.13328
660	1229	2276	10.0579	1196.89	0.105152
683	1950	2309	12.834	1851.13	0.16263
776	1739	1222	10.4485	473.462	0.041596
780	1228	1823	11.7877	929.235	0.081637
790	1052	1718	10.4485	562.309	0.049401
794	635	1487	11.3971	984.352	0.08648
804	1161	1309	7.93753	414.356	0.036403
813	633	975	5.95664	394.603	0.034668
814	471	1194	14.2848	1016.95	0.089344
819	307	1242	13.5175	1459.74	0.128244

Table S12. Proteins only identified in strain YJ56 cultured at 25 °C. Strain YJ56 expressed 171 proteins at 25 °C. The number of selected proteins for LC/MS analysis was 9 spots (bold).

SpotID	X	Y	Area	Vol	%Vol
26	973	533	7.85383	458.996	0.043229
35	711	612	10.5322	688.473	0.064842
36	739	613	11.7738	643.689	0.060624
37	566	618	11.9551	2154.26	0.202893
40	512	631	10.2672	1007.73	0.094911
43	540	631	8.35603	604.201	0.056905
46	324	642	21.5109	3160.6	0.297672
**47**	**680**	**651**	**15.2473**	**2418.01**	**0.227733**
50	955	658	7.79803	418.932	0.039456
51	991	665	8.96983	452.786	0.042644
53	401	714	13.6291	1693.23	0.159473
60	597	737	13.0851	1281.01	0.120649
61	718	739	8.48158	529.973	0.049914
66	872	754	7.68643	430.468	0.040543
71	901	783	9.06748	614.464	0.057872
77	277	812	14.3406	1049.21	0.098817
82	649	831	13.2106	1995.57	0.187947
83	777	824	9.41623	461.856	0.043499
**86**	**820**	**834**	**14.0616**	**1259.3**	**0.118603**
96	760	854	8.87218	600.114	0.05652
98	672	871	13.099	1582.74	0.149066
102	802	889	7.43533	476.015	0.044832
104	335	900	30.6202	4884.89	0.46007
**105**	**906**	**901**	**12.5271**	**1043.71**	**0.101299**
**110**	**866**	**919**	**8.73268**	**1349**	**0.127052**
113	933	921	8.25838	208.554	0.019642
115	726	932	4.29659	415.416	0.039125

117	577	942	13.9221	2154.49	0.202915
125	935	951	9.31858	452.536	0.042621
131	593	967	8.85823	1154.45	0.108729
133	990	976	7.53298	345.917	0.032579
134	715	974	10.4904	893.49	0.084151
135	1057	977	5.13359	234.976	0.022131
137	749	984	8.09098	892.784	0.084084
140	789	988	6.41699	482.655	0.045458
142	1164	985	7.01684	329.945	0.031075
**145**	**833**	**998**	**13.1688**	**2478.41**	**0.233422**
**154**	**1390**	**1018**	**15.61**	**1443.89**	**0.135989**
155	717	1026	6.83549	460.53	0.043374
159	912	1044	8.17468	281.963	0.026556
162	619	1053	5.53814	327.113	0.030808
166	830	1060	9.31858	628.988	0.05924
174	1704	1073	7.72828	562.728	0.052999
175	1276	1077	7.75618	304.333	0.028663
**177**	**663**	**1086**	**18.4419**	**3202.36**	**0.301606**
178	507	1100	23.8126	3732.75	0.351559
183	995	1097	6.83549	336.906	0.031731
184	1495	1097	12.82	749.225	0.070564
185	571	1103	11.718	1061.33	0.099958
186	875	1101	10.044	1032.7	0.097262
190	347	1114	12.8898	978.172	0.092127
192	1189	1117	9.87658	548.499	0.051659
196	1529	1149	10.1835	642.243	0.060488
201	640	1141	11.146	825.434	0.077741
205	613	1152	6.47279	183.567	0.017289
208	804	1159	10.6717	893.719	0.084173
215	944	1181	8.59318	465.008	0.043796

216	509	1182	15.6658	2959.29	0.278713
221	728	1190	12.4992	1146.77	0.108006
222	657	1191	10.2253	993.238	0.093545
225	555	1203	8.85823	1183.2	0.111437
226	687	1201	9.62548	656.965	0.061874
233	798	1216	9.12328	610.605	0.057508
238	587	1226	7.53298	888.237	0.083656
239	1492	1228	10.3788	650.333	0.06125
243	727	1230	15.889	1100.71	0.103667
247	773	1238	11.6482	801.359	0.075474
250	565	1254	9.43018	973.599	0.091696
252	1523	1252	8.28628	433.258	0.040805
263	725	1279	9.90448	750.522	0.070686
264	681	1278	12.7782	1569.79	0.147846
266	1056	1287	14.0616	666.091	0.062734
269	1272	1295	13.6152	685.739	0.064584
277	1522	1320	12.3178	479.628	0.045172
283	718	1333	9.68128	734.404	0.069168
284	537	1334	9.66733	379.558	0.035748
286	747	1339	12.9456	1145.74	0.107908
292	561	1355	9.73708	893.258	0.084129
295	415	1357	9.27673	409.315	0.03855
302	923	1371	11.1181	794.032	0.074784
303	676	1374	13.5175	1252.73	0.117985
307	1321	1378	8.35603	369.883	0.034837
311	386	1398	15.4845	805.049	0.075821
313	679	1399	17.4235	1863.41	0.1755
314	1115	1407	11.425	926.32	0.087243
317	1001	1400	13.1827	837.333	0.078862
333	831	1445	11.2716	1108.35	0.104387

337	451	1445	11.5506	820.669	0.077293
343	879	1464	7.82593	547.997	0.051612
344	616	1468	9.47203	1504.25	0.141674
345	249	1471	20.9808	810.914	0.076374
351	558	1478	8.52343	780.71	0.073529
354	1364	1485	6.66809	311.656	0.029353
355	753	1486	9.16513	1665.57	0.156868
358	440	1496	18.5535	2557.11	0.240835
359	662	1499	6.29144	265.369	0.024993
361	544	1505	11.7319	1022.13	0.096267
369	1446	1520	12.3457	764.054	0.07196
375	723	1532	11.3692	1131.4	0.106558
377	115	1548	20.632	2031.06	0.19129
383	672	1554	9.17908	717.929	0.067616
384	714	1555	12.4852	852.172	0.08026
388	311	1564	14.2011	1542.73	0.145298
393	577	1573	8.88613	445.821	0.041988
394	682	1572	9.01168	775.088	0.073
396	927	1576	12.4573	847.475	0.079817
399	980	1587	16.3633	674.032	0.063482
402	892	1588	8.56528	168.853	0.015903
406	712	1599	17.3259	1666.45	0.15695
407	524	1590	11.0205	1150.26	0.108334
409	306	1594	8.74663	688.975	0.064889
413	1687	1602	12.82	347.712	0.032748
420	1082	1609	15.0241	816.115	0.076864
424	606	1627	15.6798	1707.43	0.16081
429	1698	1633	9.15118	207.439	0.019537
435	443	1640	11.0065	722.919	0.068086
440	593	1652	16.7121	1515.57	0.14274

441	1683	1651	11.6761	534.688	0.050358
**445**	**645**	**1674**	**19.8369**	**3619.54**	**0.340897**
450	971	1679	9.02563	576.036	0.054252
452	1178	1681	10.9926	860.336	0.081028
456	732	1699	14.1453	449.199	0.042307
463	553	1718	19.8927	4541.8	0.427757
465	618	1727	20.5344	4774.47	0.44967
474	1018	1763	10.4625	738.177	0.069523
475	1052	1765	13.8244	1097.23	0.10334
490	889	1826	11.3692	944.873	0.08899
491	727	1825	9.16513	480.2	0.045226
496	572	1833	13.7128	1300.37	0.122472
498	618	1846	11.5924	1641.23	0.154575
503	855	1863	13.0572	805.555	0.075869
512	953	1913	12.0109	587.154	0.0553
524	551	1972	12.6247	989.262	0.093171
529	699	1985	14.5219	1382.74	0.130229
531	1166	1989	16.5726	593.728	0.055919
532	498	1992	11.704	1526.84	0.143801
533	597	2000	7.82593	863.685	0.081344
544	589	2039	17.7304	2107.74	0.198512
**569**	**675**	**2111**	**23.7289**	**4279.58**	**0.403061**
574	485	2112	7.95148	766.844	0.072223
583	484	2132	8.21653	479.969	0.045205
623	452	2235	10.9368	3218.94	0.303167
652	1475	2271	11.9691	994.256	0.093641
673	578	2315	11.2716	1768.44	0.166555
746	874	505	7.93753	397.532	0.037441
747	1238	836	9.16513	473.141	0.044562
750	1386	1210	10.4485	389.888	0.036721

753	839	934	9.16513	483.185	0.045508
755	885	939	9.70918	798.092	0.075166
756	995	848	9.16513	439.801	0.041421
757	858	777	6.73784	288.75	0.027195
758	887	1162	7.93753	419.155	0.039477
759	863	1128	7.93753	462.246	0.043535
760	769	1286	9.16513	574.753	0.054132
761	1112	924	6.87734	320.687	0.030203
762	1127	934	5.56604	184.67	0.017393
766	1416	1605	11.7877	511.099	0.048137
767	528	1786	11.7877	1041.45	0.098086
770	266	1193	17.3119	2513.98	0.236772
771	945	1764	13.3501	812.879	0.076559
773	862	1816	12.4015	764.584	0.07201
774	1051	1584	13.3501	756.144	0.071215
775	1037	1632	10.4485	488.361	0.045995
777	829	1844	9.70918	680.312	0.064073
778	905	1305	10.9368	1037.1	0.097676
781	1007	591	9.16513	310.122	0.029208
782	1232	1016	9.16513	513.233	0.048338
783	1274	1404	12.1783	780.626	0.073521
784	413	1280	11.7877	1326.17	0.124902
785	458	1312	13.0293	1381.1	0.130075
787	789	1055	6.82154	257.907	0.02429

Figure S1. Culture-independent bacterial communities in Antarctic soil using Oxford Nanopore sequencing tools. The same amounts of soil samples (1 g) were inoculated in an R2A liquid at 13 °C and 30 °C, respectively. Incubated soil samples were harvested at specific periods (0, 7, 14, and 28 days) to estimate biomass (DNA) and cell density. **a, c** The bacterial biomass was estimated using DNA quantification and the bacterial community was analyzed using the CLC Genomics Workbench ver.21 (Qiagen, Germany) software. **b, d** Cell density was measured using the CFU assay. *N. D: not detected.

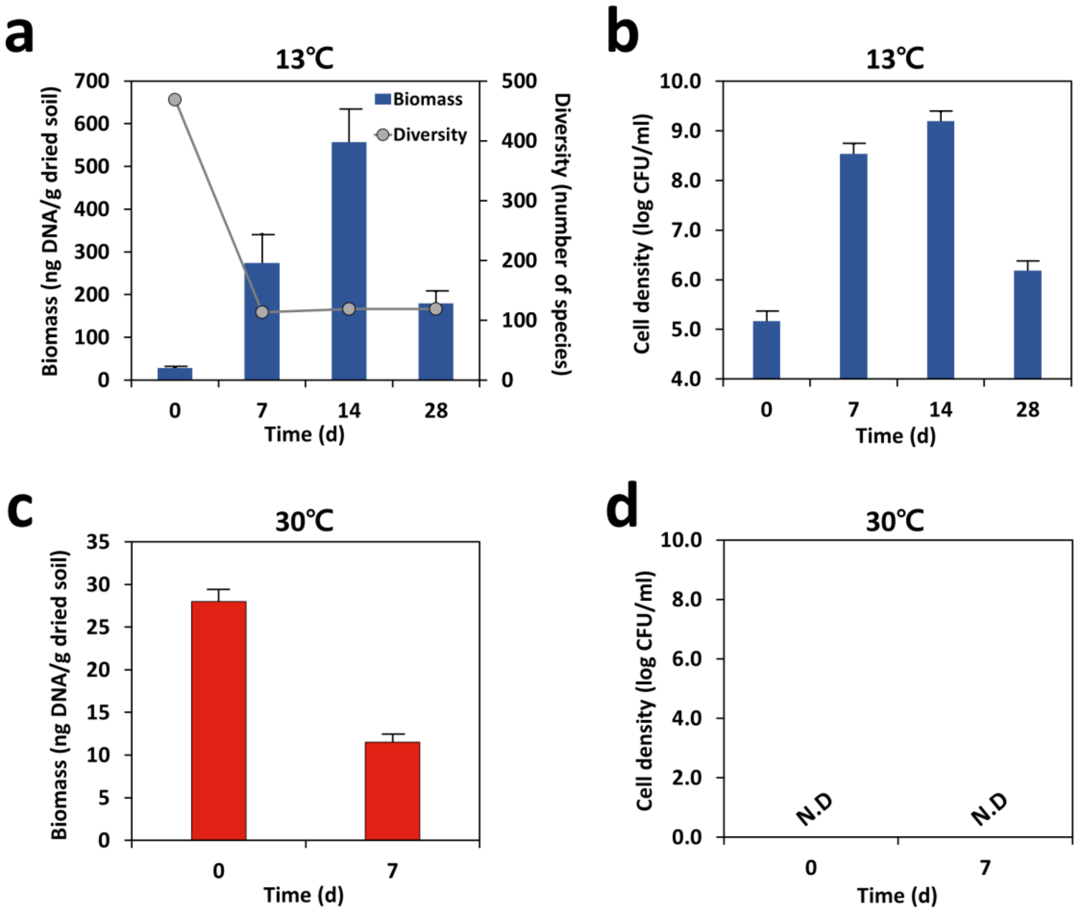

Figure S2. Culture-independent bacterial communities in Antarctic soil using T-RFLP analysis. **a** and **b** T-RFLP analysis for assessing culture-independent bacterial communities in Antarctic soil. The T-RFLP analysis was performed by using the restriction enzymes *MspI* and *Rasl.* The soil samples for the analysis were incubated in an R2A liquid medium at 13 °C and harvested at specific periods (0, 7, 14, and 28 days). **a** The peak of strain YJ56 was shown in 160 to 163 bps of fragment lengths distinguished by a red line. **b** The peak of strain YJ56 was shown in 454 to 457 bps of fragment lengths distinguished by a red line. The peaks identified by species were indicated by using arrows.

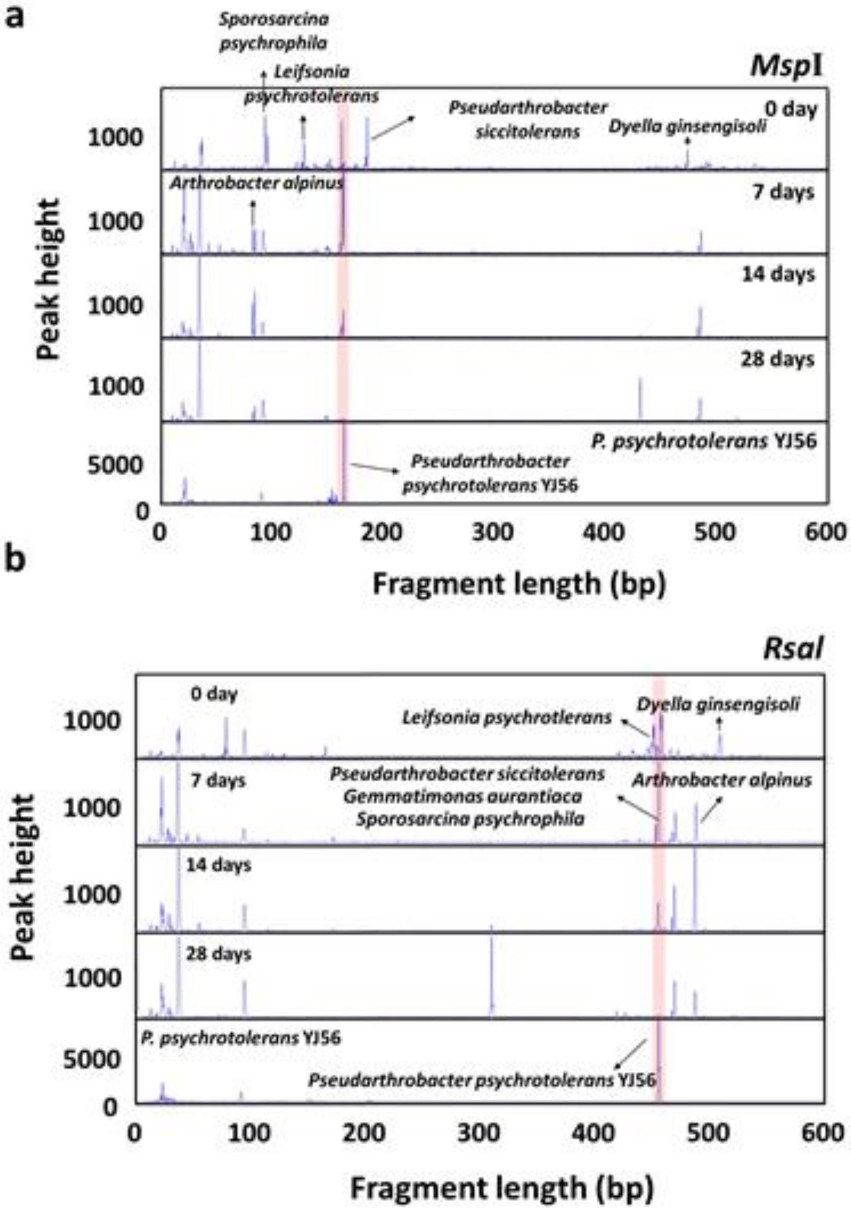

Figure S3. Growth test of psychrophilic bacteria isolated from Antarctic soil. A total of the 60 strains were inoculated in R2A medium and grown at 13 °C for 5 days. The OD_{600} values of each strain were measured by the spectrometer (24-hour unit intervals). Mesophilic *Escherichia coli* ATCC 25922 was used as a negative control. The red dotted line indicates OD_{600} of 3.0.

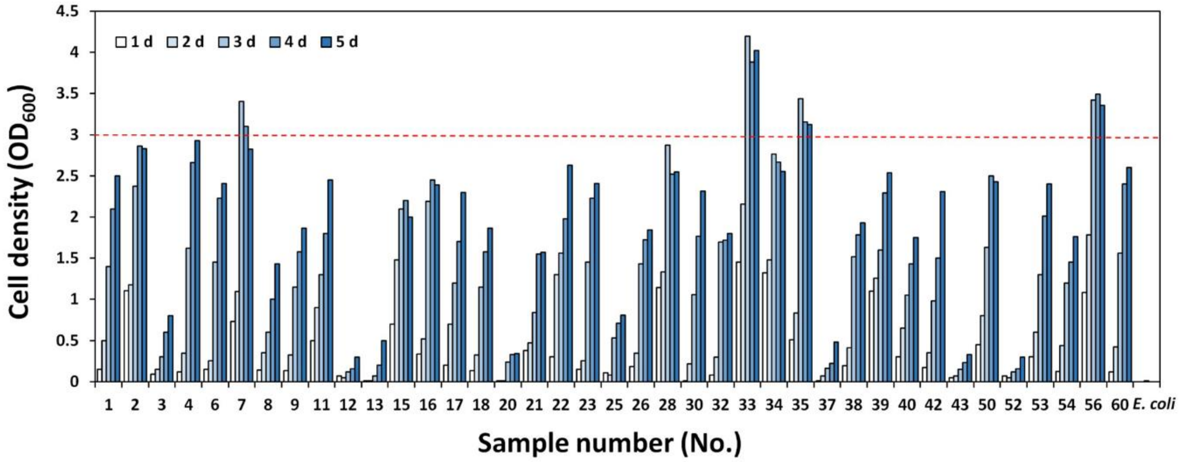

Figure S4. Phase-contrast microscopic observation of strain YJ56 cultured grown on an R2A liquid medium at 13℃ (**a** and **b**) and 25℃ (**c** and **d**) until the exponential phase (OD_{600}: 1.0) using a confocal laser scanning microscope (Carl Zeiss, Germany) with 5 µm of the scale bar. Red and yellow arrows indicate cell lengths and widths of strain YJ56, respectively.

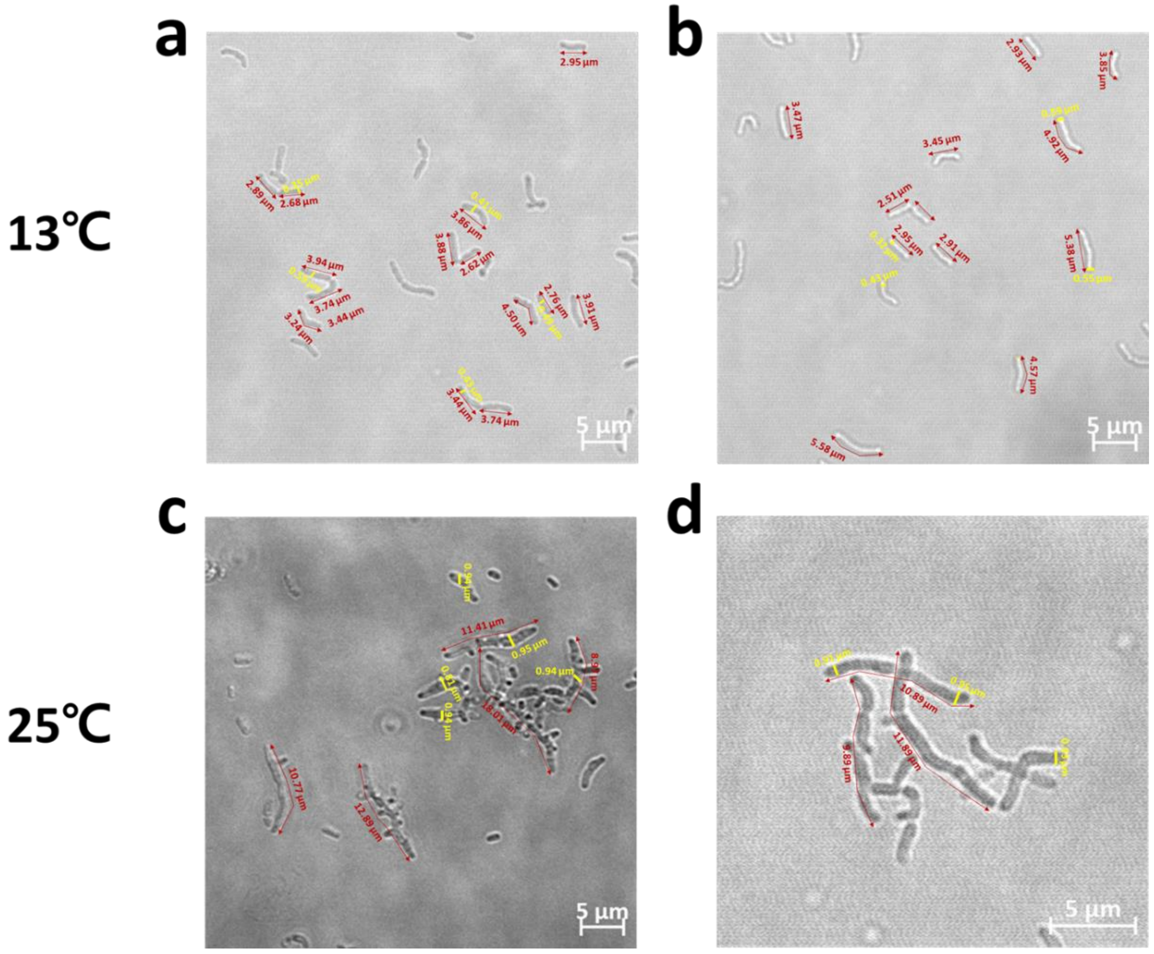

Figure S5. Comparative genomic analysis of *P. psychrotolerans* YJ56 and other *Pseudarthrobacter* species. **a.** The “A” in the yellow box represents the site with relatively abundant *rimJ* gene copies among specific contained genomic sequences in strain YJ56. **b.** Venn diagram represents the distribution of shared and unique gene clusters by OrthoVenn2 analysis.

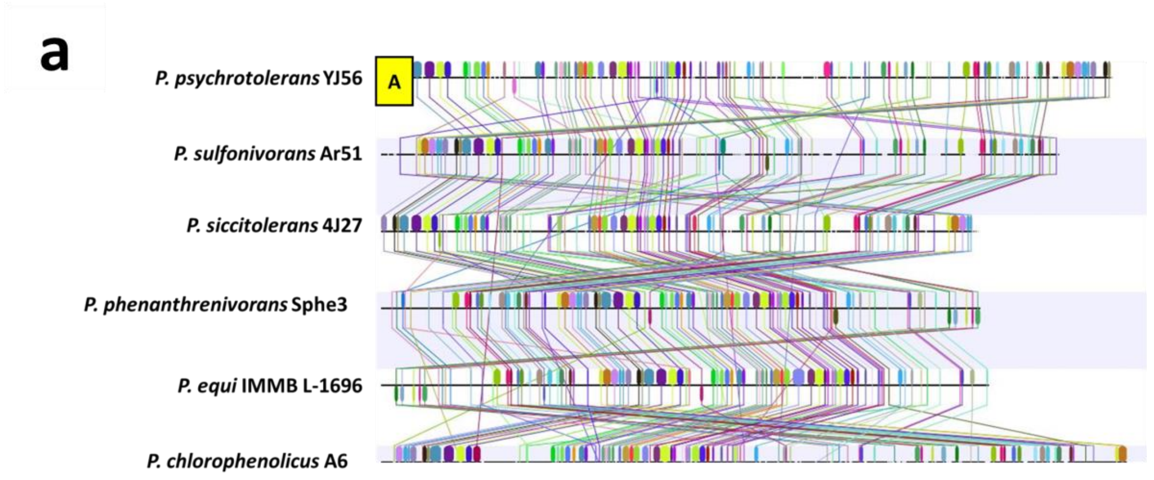

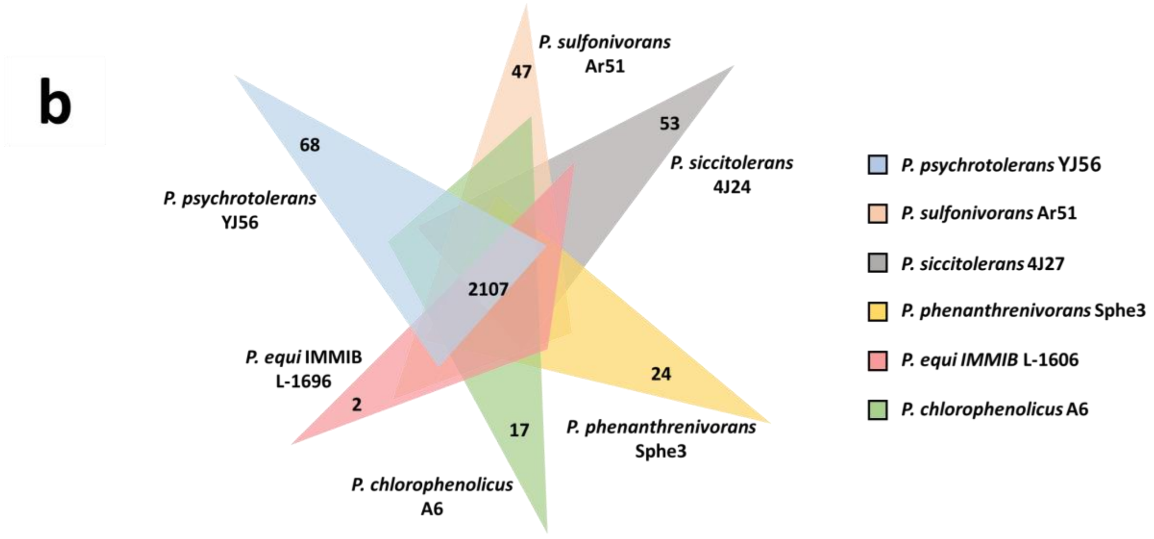

Figure S6. 2-DE image analysis of proteins expressed from strain YJ56 cultured at 13 °C or 25 °C. The images were analyzed with Image Master Platinum 5.0 image analysis program. Green and red spots indicate paired (n: 238) and non-paired spots (n: 265), respectively.

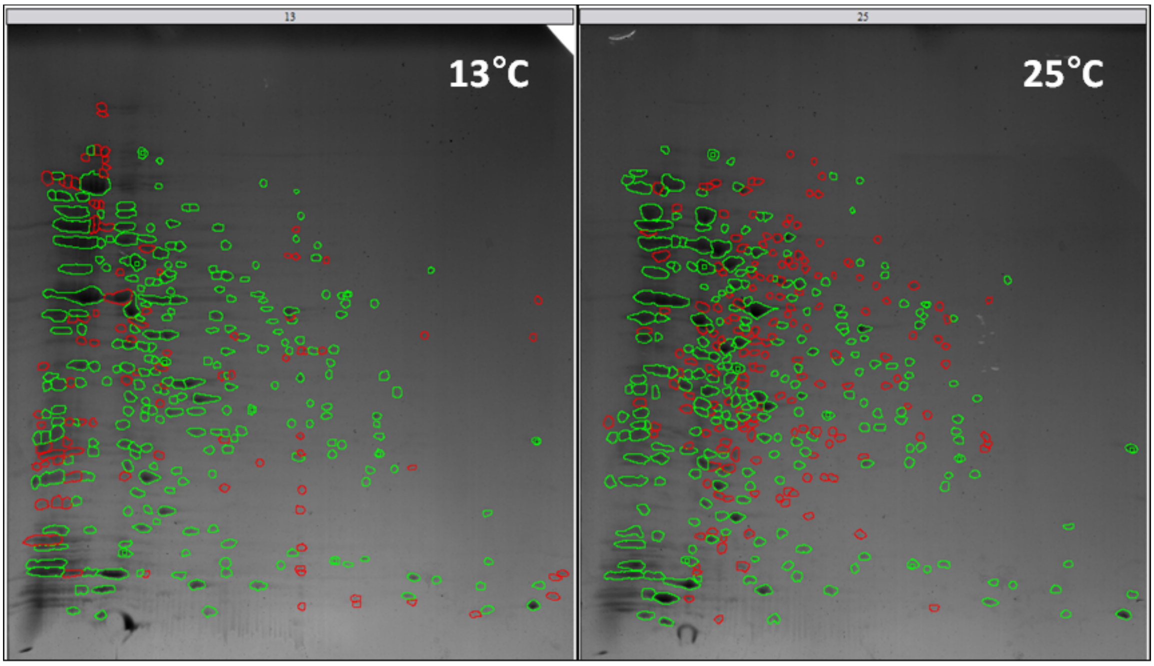

green=paired spots / red=non-paired spots

Figure S7. 2-DE analysis for identifying different protein expressions in strain YJ56 cultured at 13 ℃ or 25 °C. Proteins with increased abundance in strain YJ56 cultured at **a** 13 ℃ or **b** 25 °C. The total number of selected spots was five and ten at 13 ℃ and 25 °C, respectively. Proteins only identified in strain YJ56 cultured at **c** 13℃ or **d** 25 °C. The total number of selected spots was four and nine for each temperature condition (13 ℃ and 25 °C), respectively. Selected proteins were indicated with yellow, white, and green triangles. Red circles are a standard point of arrangement for gel images.

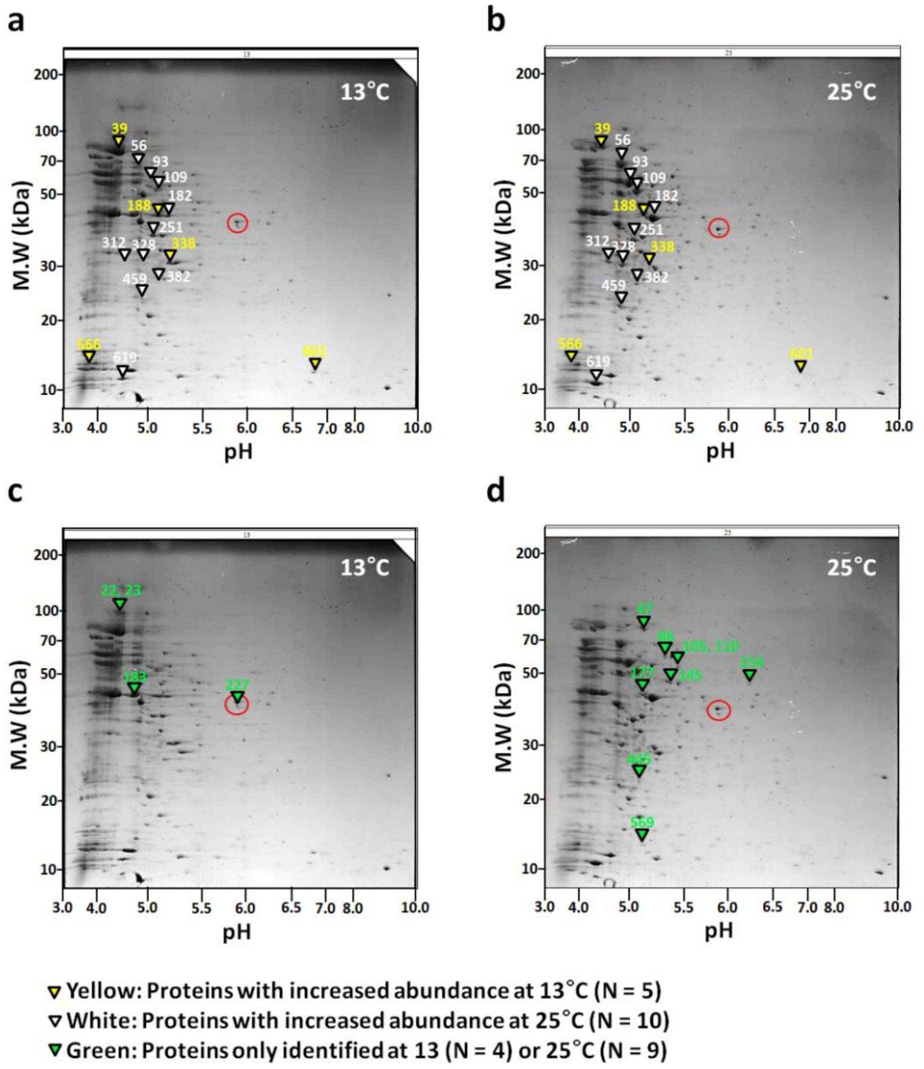

Figure S8. Fatty acid proportions of strain YJ56 according to temperature conditions. Saturated fatty acids were identified $C_{9:0}$, $C_{14:0}$, $C_{16:0}$, $C_{18:0}$, iso-$C_{14:0}$, iso-$C_{15:0}$, anteiso-$C_{15:0}$, anteiso-$C_{15:0}$ A, iso-$C_{16:0}$, iso-$C_{15:0}$ H, and anteiso-$C_{17:0}$. Unsaturated fatty acids were identified $C_{14:1}\omega 5c$, anteiso-$C_{17:1}\omega 5c$, $C_{8:0}$3-OH, iso-$C_{17:0}$3-OH, and $C_{16:1}\omega 7c$ and/or $C_{16:1}\omega 6c$. The total ratio of saturated and unsaturated fatty acids at each temperature was described as a percentage (%).

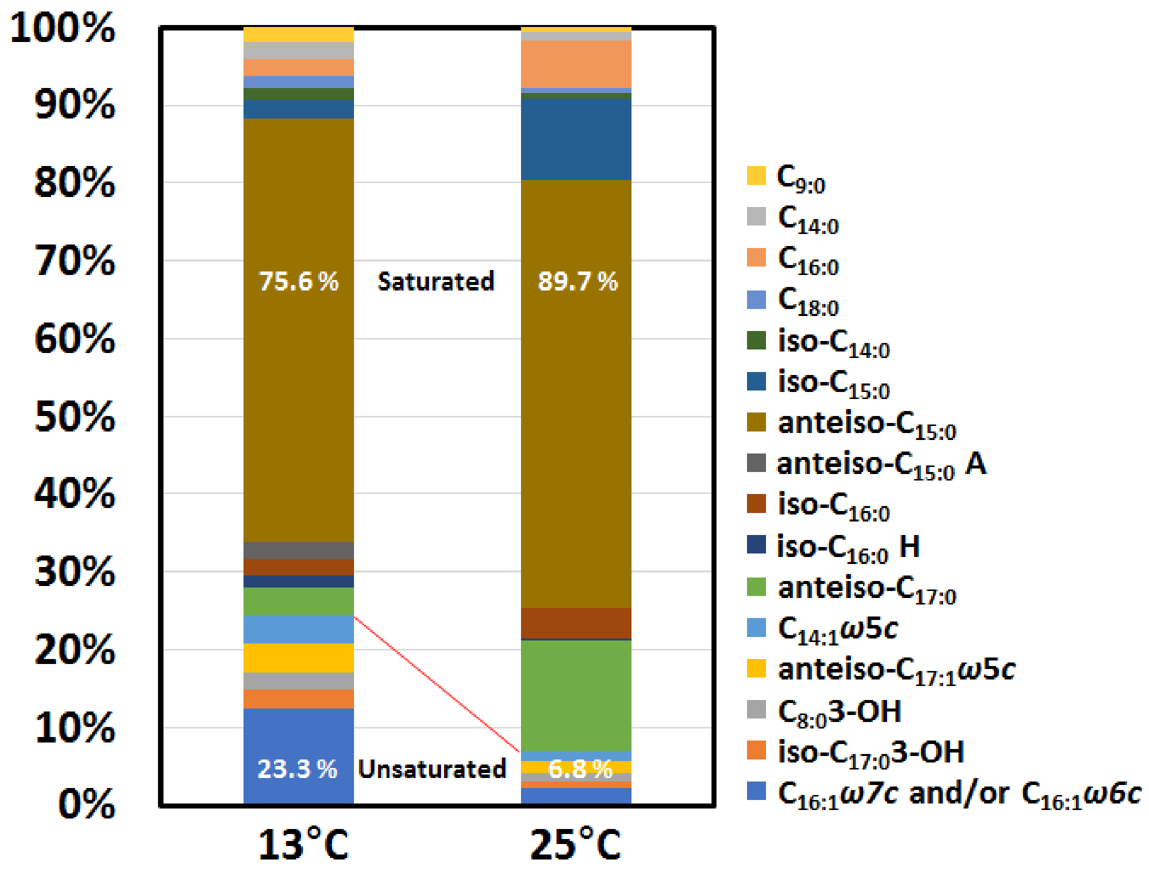

Supplement: Supplementary file 1 — Supplementary Information. [file 41598_2023_42179_MOESM1_ESM.pdf]
